# Food-Based Electronics: Revisiting β‑Carotene Organic Transistors

**DOI:** 10.1021/acsami.5c23614

**Published:** 2026-02-23

**Authors:** Alberto D. Scaccabarozzi, Elena Feltri, Pierluigi Mondelli, Pietro Rossi, Francesca Pallini, Antonella Treglia, Annamaria Petrozza, Luca Beverina, Giuseppe Mattioli, Jaime Martin, Alessandro Luzio, Mario Caironi

**Affiliations:** † Center for Nano Science and Technology, 403543 Istituto Italiano di Tecnologia, Via Rubattino, 81, Milano 20134, Italy; ‡ Department of Physics, Politecnico di Milano, Piazza Leonardo da Vinci, 32, Milano 20133, Italy; § Department of Materials Science, 9305 University of Milano-Bicocca, Via R. Cozzi, 55, Milano 20126, Italy; ∥ Consiglio Nazionale Delle Ricerche (CNR), 204549 Istituto di Struttura della Materia (ISM), Strada Provinciale, 35d/9, Montelibretti 00010, Italy; ⊥ 16737 Universidade da Coruña, Campus Industrial de Ferrol, CITENI, Esteiro, Ferrol 15403, Spain

**Keywords:** organic electronics, edible electronics, organic transistors, sustainable electronics, bioderived semiconductors

## Abstract

Edible electronics offer a unique platform for developing devices made entirely from food-based materials that can be safely digested or excreted without environmental concerns. Yet, identifying semiconductors that are both food-based and capable of supporting efficient charge transport remains a challenge. In this work, we show that this hurdle can be overcome by applying structure–property insights developed in organic electronics to natural compounds, revealing how a material previously discarded for electronic applications and largely present in vegetables, β-carotene, can be tuned into a viable semiconductor. Beyond its implications for edible electronics, this approach also highlights the broader potential of renewable, nature-derived materials as building blocks for sustainable technologies.

## Graphical abstract

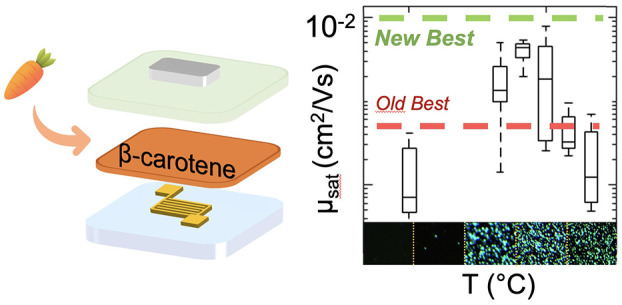

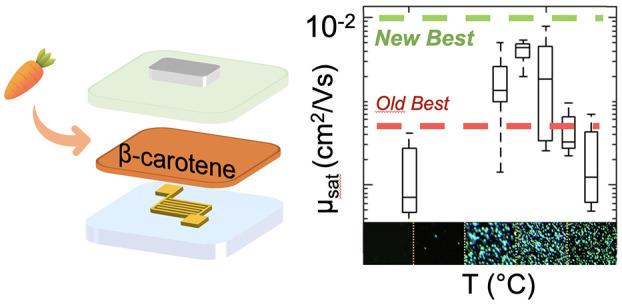

## Introduction

Edible electronics is an emerging field that envisions devices composed entirely of food-derived, digestible materials, hence extending beyond traditional ingestible electronics, which are instead primarily designed to ensure safety during transit through the body.^1^ Indeed, unlike conventional systems, fully edible platforms would not only be safe for consumption but would also be treated by the body like food: partly absorbed for their nutritional value and safely excreted for the rest, eliminating the need for recollection and reducing health and safety risks, as well as the need for hospitalization. This approach unlocks new opportunities for safe and noninvasive biomedical monitoring, smart food packaging for advanced food quality control and food waste reduction, environmentally friendly electronic systems, as well as sensing and computation in future edible robots.^2^ Moreover, edible materials can be potentially derived from renewable biological sources, reducing the reliance on petrochemicals and enabling their integration into circular material flows. As such, edible electronics align closely with global efforts toward circular technologies and environmentally conscious design.

Our group has recently reported several examples of edible electronic components, including edible electrolytes,^3^ conductive pastes,^4,5^ honey-gated circuits,^6^ edible batteries and supercapacitors,^7–9^ and edible sensors.^10,11^ Despite the growing interest in this field, a key challenge remains: the development of edible semiconductors, which are essential for enabling active electronic components such as transistors. To address this, we have recently proposed Copper Phthalocyanine (CuPc) as a viable candidate.^12,13^ CuPc is widely used as a pigment in toothpaste, with no safety issues emerging after several years of observation. Our studies have shown that it is routinely ingested in far greater amounts during toothbrushing than would ever be present in our devices, supporting its safe use in edible electronics. Nevertheless, although deemed safe, CuPc remains a synthetic cosmetic pigment. In parallel, biocompatible polymers such as Poly­(3-hexylthiophene-2,5-diyl) P3HT have been investigated in this context, with recent studies by Coco et al. addressing short-term biological responses, while questions regarding actual edibility and long-term applicability remain open.^14^ In contrast, nature offers a rich palette of colorful, conjugated dyes that, at least in principle, combine optoelectronic properties, edibility, and sustainability. However, despite these appealing features, most natural dyes fall short in terms of stability, processability, or charge transport, limiting their practical application in electronics. Among them, carotenoids, a class of naturally occurring conjugated organic molecules found in many food sources, have emerged as particularly promising candidates. Indeed, carotenoids exhibit a linear extended π-conjugated polyene chain, responsible for their semiconducting nature and remarkable optoelectronic properties,^15^ playing a fundamental role in biological systems.^16^ For instance, they function as light harvesters in photosynthesis, efficiently transferring energy to chlorophyll molecules. Additionally, they serve a photoprotective role, dissipating excess energy through nonradiative decay mechanisms, play a role in charge transport mechanisms, and are critical in biological photodetection, i.e., in vision, where retinal, which is a carotenoid-derived chromophore, acts as the light-sensitive component.^17^ Moreover, thanks to their antioxidant properties, they are not simply edible but also possess significant nutritional value, being Vitamin A precursors, and contribute to the maintenance of human health.^18–21^

Beyond their relevance to edible electronics, the natural origin of carotenoids positions them as a sustainable alternative to conventional organic electronic materials, which are often petrochemical-derived and achieved via multistep, unsustainable synthesis.^22,23^ Indeed, unlike usual synthetic semiconductors, carotenoids can be extracted from natural sources,^24–26^ reducing the reliance on fossil-fuel-based materials and avoiding the employment of complex synthetic routes and hazardous solvents, key features for a circular, sustainable electronics platform.

Among this class of materials, β-carotene has been extensively studied as a reference carotenoid due to its symmetrical structure, featuring a hydrocarbon chain devoid of additional elements, reminiscent of prototypical organic semiconductors such as polyacetylene, with 11 conjugated double bonds and two cyclohexene rings as terminal groups (Figure 1a). Although β-carotene has previously been used as the active layer in organic field-effect transistors (OFETs) in the form of thin films, it was generally classified as a low-mobility (μ ≈ 10^–4^ cm^2^/(V s)), unstable semiconductor, and was subsequently largely overlooked.^27–29^ However, these early studies preceded the significant advances in organic electronics that now enable a more refined understanding and control of structure–property relationships.^30^ Building on this knowledge, in this work, we revisit β-carotene and explore its electronic properties in depth, establishing a direct correlation between microstructure, charge transport, and stability. Thereby, we show that careful microstructural engineering, enabled through precise solvent selection and controlled annealing conditions, can significantly enhance the charge transport properties of β-carotene thin films, yielding charge carrier mobilities exceeding 10^–2^ cm^2^/(V s), far surpassing previously reported values. The same approach also mitigates previous concerns regarding stability, showing that these limitations can be effectively addressed through appropriate processing strategies. Our findings suggest that β-carotene, when properly processed, can serve as an efficient semiconducting material, furthering the realization of edible, sustainable electronic devices.

**Figure 1:**
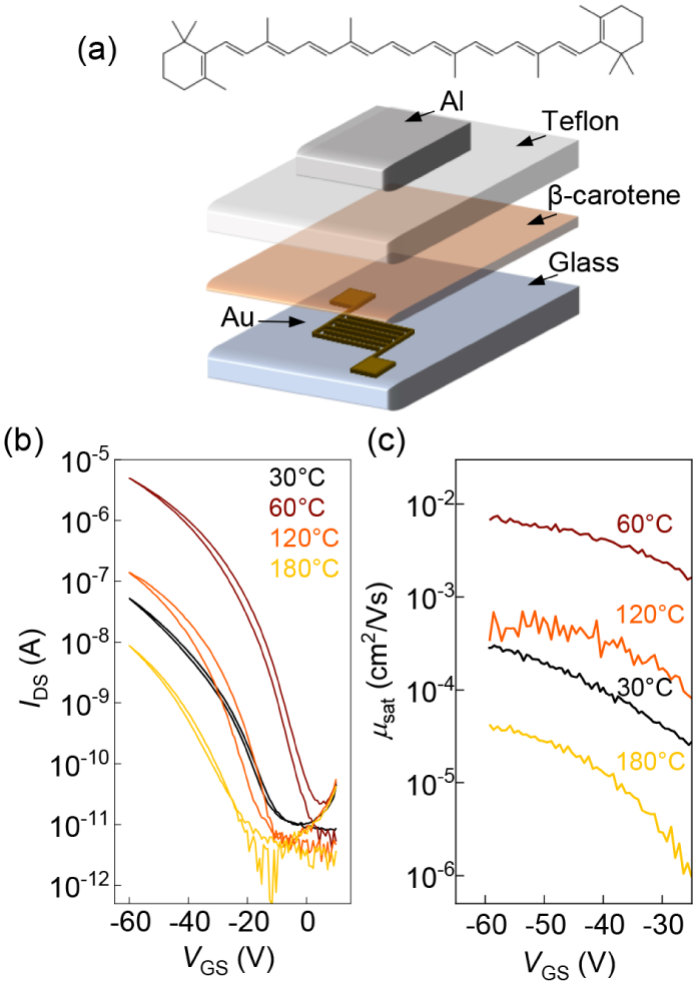
(a) Chemical structure of β-carotene and illustration of the TGBC device architecture. (b) Representative transfer curves and (c) corresponding charge carrier mobilities for TGBC transistors based on β-carotene cast from THF measured in the saturation regime (*V*_DS_ = −60 V) for different annealing temperatures, as indicated. Channel length *L* = 10 μm, channel width *W* = 2 mm.

## Results and Discussion

We focused our investigation on solution processing, as it is a versatile and widely adopted approach in organic electronics, allowing fine control over the structural and optoelectronic properties of thin films. Among various solvents, tetrahydrofuran (THF) was selected as it is among the best solvents for β-carotene, ensuring high solubility,^31,32^ which allows proper solution formulation and the formation of homogeneous thin films suitable for device fabrication. In addition, the low boiling point of THF (66 °C) facilitates efficient solvent removal during film processing following principles commonly adopted in food and pharmaceutical manufacturing to minimize residual solvents. We then selected a standard top-gate, bottom-contact configuration (TGBC) (Figure 1a) to fabricate field-effect transistors and assess the transport properties of our β-carotene films. Solutions (5 mg/mL) were spin-cast onto glass substrates with previously patterned gold microelectrodes, while a solution-processable Teflon-based layer was cast on top, serving as a dielectric. Finally, aluminum was thermally evaporated onto the structure to complete the TGBC device architecture.

Transistors based on as-cast β-carotene films from THF, labeled as “30 °C” to reflect the postdeposition processing temperature, show p-type field-effect behavior with poor performance (Figure 1b-c). Transfer curves in the saturation regime exhibit low on-currents (*I*_on_ < 10^–7^ A) and threshold voltages shifted toward negative voltages (*V*_t_ = −20 V). Output characteristics show nonidealities, including S-shaped curves (Figure S1), indicative of charge injection barriers. Charge-carrier mobility (μ) shows a pronounced gate voltage modulation, reflecting the nonlinearity of the *I–V* curves (i.e., the *I*_DS_(*V*_GS_) and |*I*_DS_|^1/2^(*V*_GS_) dependences in the linear and saturation regimes, respectively), with μ_sat_ ≈ 10^–4^ cm^2^/(V s) extracted at *V*_GS_ = −60 V, a value consistent with previous reports from the literature.^27^ Overall, the electrical characteristics suggest a relatively poor charge transport with a non-negligible contact resistance and high trap density. Interestingly, a thermal annealing at relatively low temperatures (*T* ≈ 60 °C), applied to as-cast films, leads to a significant improvement in device performance, with *I*_DS_ increasing by nearly 2 orders of magnitude (Figure 1b,c). As a result, the corresponding charge-carrier mobility in saturation μ_sat_ approaches 10^–2^ cm^2^/(V s), with an improved linearity of |*I*_DS_|^1/2^(*V*_G_) (Figure S1) and thus, reduced dependence of μ_sat_ on the gate voltage. In contrast, an annealing at higher temperatures (*T* > 60 °C) resulted in a progressive decline in device performance, with a reduction of *I*_on_ and μ (Figure 2a), following a rather complex trend: an initial and progressive drop up to 90 °C (Figure S2), followed by a partial recovery at 120 °C, and a further decrease at higher temperatures (Figure S3).

**Figure 2:**
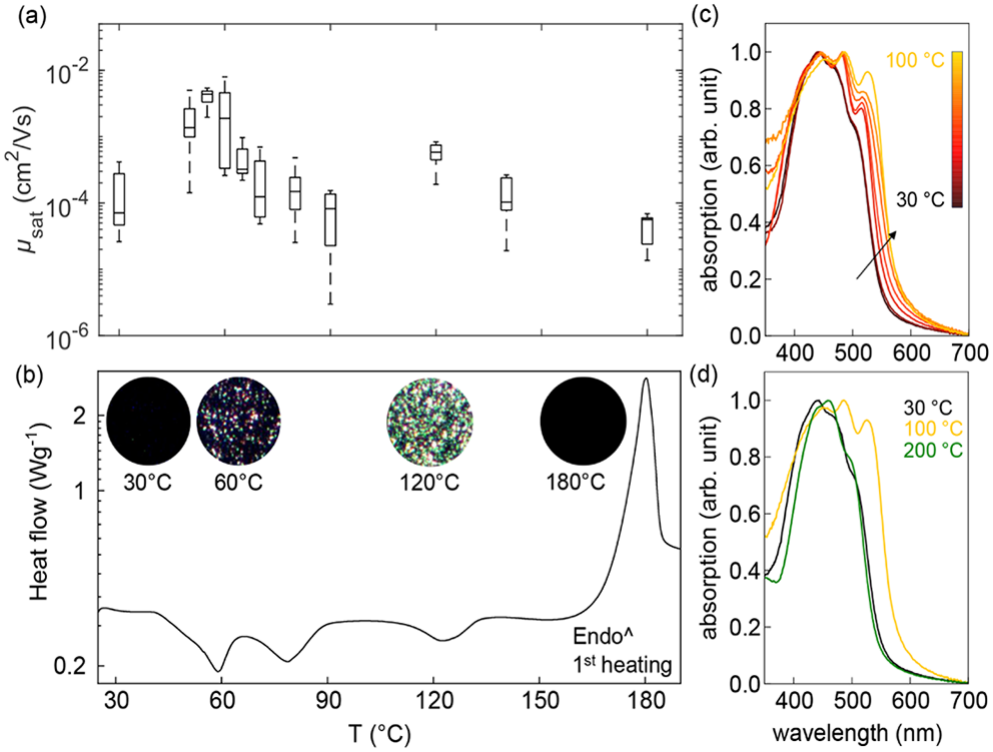
β-carotene was cast from THF. (a) Charge carrier mobilities extracted in the saturation regime as a function of annealing temperature. (b) Differential Scanning Calorimetry (DSC) thermographs—first heating scan—with polarized optical microscopy (POM) images shown in onset. (c, d) Normalized UV–vis absorption spectra as a function of annealing temperature.

To shine light on the relationships between annealing and device performance, we performed a thorough characterization of the β-carotene thin films. We employed Differential Scanning Calorimetry (DSC, Figure 2b) to provide insights into the thermal properties of β-carotene and thus investigate whether phase transitions occur upon annealing. Interestingly, the first heating scan of β-carotene processed from THF reveals three distinct exothermic peaks at approximately 60 °C, 80 °C, and 120 °C, while a sharp endothermic peak is observed at *T* ≈ 180 °C, corresponding to melting. Melting is known to be accompanied by isomerization processes that prevent any recrystallization upon cooling, as previously reported (Figure S4).^33^ Indeed, the first cooling scan is essentially featureless, and so are further cycles, apart from the presence of a feature with an inflection point at 39 °C, probably related to a glass transition (*T*_g_). Thus, the β-carotene cast from THF undergoes three exothermic transitions before melting, which leads to a further amorphous microstructure accompanied by isomerization.

With the aim of assessing the structural changes associated with the thermal transitions, we employed a combination of Grazing Incident Wide Angle X-ray Scattering (GIWAXS), Atomic Force Microscopy (AFM), optical microscopy, and UV–vis spectroscopy. UV–vis absorption spectra of β-carotene films cast from THF reveal a progressive sharpening of spectral features upon annealing and increased intensity at higher wavelengths (Figure 2c), suggesting increased molecular order and enhanced intermolecular interactions. In contrast, at temperatures above 150 °C, the spectra lose definition (Figure 2d), with a drop in absorption at high wavelengths. The exact spectral assignment is rather complex, as the UV–vis spectra of carotenoids have been variably interpreted as arising from the formation of both H- and J-aggregates.^34–37^ Nevertheless, the increased intermolecular interactions and enhanced excitonic coupling suggest a structural evolution accompanied by an increased order, which is then lost upon further raising the annealing temperature. This interpretation is in agreement with UV–vis spectra calculated using time-dependent DFT in the case of simplified models of crystalline and amorphous β-carotene aggregates (Figure S5). Accordingly, as-cast films appear featureless when observed under optical microscopy and remain dark when analyzed via polarized light (Figure 2b onset and Figure S6). Consistently, 2D-GIWAXS patterns exhibit a broad halo (Figure 3e), while AFM images appear highly uniform and featureless (Figure 3a). These data clearly indicate that β-carotene films, as cast from THF solutions, are largely disordered. THF is indeed a low boiling point (66 °C) solvent, and as such, the fast solvent removal during spin coating does not allow sufficient time for crystallization. Thus, films solidify into a kinetically trapped amorphous state. On the other hand, upon annealing, in correspondence with the first exothermic transition observed in DSC, with onset at 45 °C and peaking at 60 °C, films become birefringent, showing small bright features, when observed under polarized optical microscopy (Figure 2b onset and Figure S6). GIWAXS patterns show multiple diffraction peaks, especially in the high-*q* region (Figure 3f), and AFM images show a topography made of small round features, besides the formation of sparse and coarser aggregates (≈1 μm) (Figure 3b and Figures S7, S8). Thus, upon thermal annealing, films undergo a crystallization process at temperatures well below their melting point, i.e., cold crystallization. Such behavior is characteristic of many organic semiconductors,^38,39^ as upon heating, molecular mobility increases sufficiently to allow nucleation and growth, hence crystallization. This transition is particularly relevant for charge transport, as it is well established that, in molecular semiconductors, charge-carrier mobility is strongly influenced by crystallinity and molecular arrangement.^40,41^ In our case, the emergence of microstructural order upon annealing leads to more efficient intermolecular charge transfer, explaining the two-orders-of-magnitude increase in mobility observed in field-effect measurements. As the annealing temperature increases beyond 60 °C, GIWAXS patterns show a progressive increase in peak intensity, number of diffractions, and anisotropy, suggesting a further enhancement in microstructural order (Figure 3g and Figures S9, S10). Notably, a Scherrer-type analysis (see Supporting Information) indicates that the crystalline coherence length remains essentially unchanged upon annealing (Table S1), suggesting that thermal treatment primarily increases the fraction and texture of the ordered material rather than promoting extended coherent crystal growth. Nevertheless, higher crystallinity does not necessarily translate into optimal charge transport. Since charge carriers percolate along the semiconductor–dielectric interface, achieving efficient transport also requires a smooth, continuous topography. Increased surface roughness, the formation of defective, disconnected crystallites, and abrupt grain boundaries can hinder charge percolation, even in the presence of high molecular order. Consistently, AFM images show a progressive coarsening of the film topography with the formation of larger, three-dimensional crystallites and structural discontinuities, disrupting the previously interconnected crystalline network (Figure 3c and Figure S7). This phenomenon is probably associated with the reported high nucleation site density of β-carotene and its tendency to fast formation of microcrystals.^42^ As a result, OFETs exhibit a drop in performance with a marked decrease in *I*–*V* ideality and charge carrier mobility. These adverse effects peak at around 90 °C, coinciding with a distinct transition just beyond the second exothermic peak observed in DSC, preventing proper device functionality, especially at longer channel lengths (Figure 2a and Figure S3). A subsequent transition emerges near 120 °C, where AFM images reveal increasingly well-defined crystallites, forming a highly ordered terraced morphology (Figure 3c). Concurrently, GIWAXS patterns display sharp increases in crystallinity and anisotropy, indicating enhanced molecular ordering (Figure 3g). Despite these improvements, the resulting crystalline domains remain only partially interconnected, limiting the full recovery of device performance. Finally, when films are annealed at *T* > 150 °C, diffraction peaks disappear (Figure 3h), POM images become dark, and AFM images become featureless (Figure 3d), in agreement with the vitrification upon melting observed in DSC.

**Figure 3:**
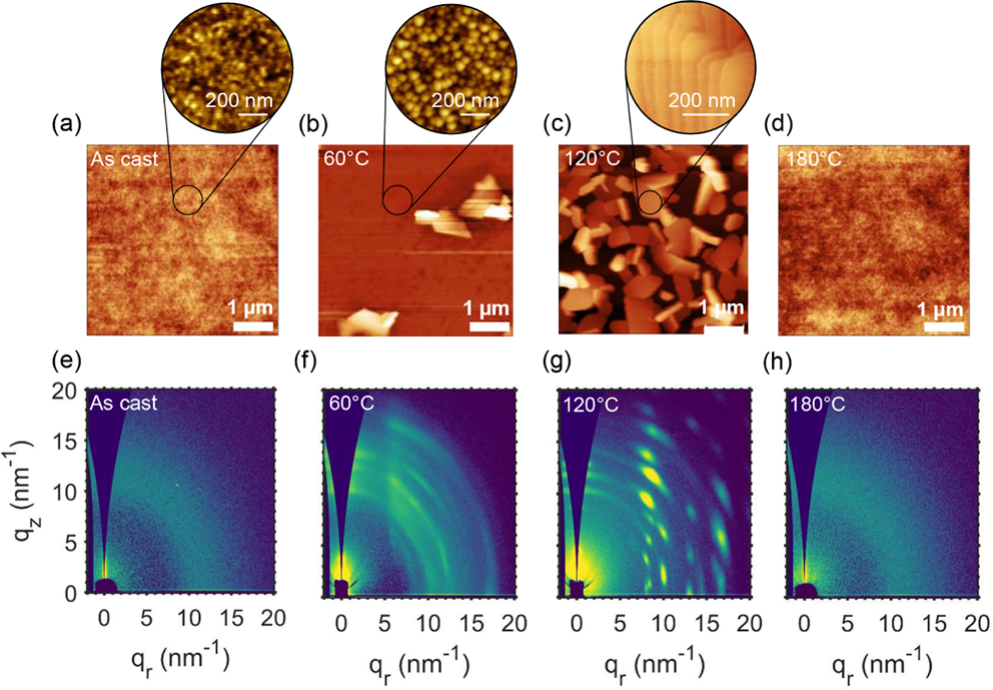
Atomic force microscopy (AFM) images (top panel) and 2D GIWAXS patterns (bottom panel) of β-carotene thin films deposited from THF. As cast (a, e), 60 °C (b, f), 120 °C (c, g), and 180 °C (d, h).

Thus, the charge carrier mobility decreases again. In summary, the high solubility of β-carotene in THF, combined with the low boiling point of the solvent, results in disordered as-cast films, whose microstructure and thus electronic properties can be tuned through annealing, yielding devices with improved performance and charge carrier mobilities approaching 10^–2^ cm^2^/(V s).

Building on the insights gained from THF-processed films, we sought to further explore the structure–property relationships by investigating other solvent systems. Indeed, it is well established how solvent selection can guide the crystallization of molecules from solution and how it plays a crucial role in crystal engineering, as it influences the solution thermodynamics, crystallization kinetics, and ultimately the size, shape, and topography of the resulting crystals.^43^ In fact, this approach has been widely employed in organic electronics to control the structural and morphological properties of the films and, ultimately, improve device performance. Thus, we selected anisole, as it exhibits markedly different properties when compared to THF, with a significantly higher boiling point (154 °C versus 66 °C), lower polarity, and reduced solubility for β-carotene. For instance, freshly prepared anisole solutions, filtered to remove any undissolved particles, initially appear clear; however, over time (a few hours), they develop visible nucleation sites, eventually forming crystals observable to the naked eye (Figure S11). Furthermore, unlike many traditional solvents used in organic electronics (*e.g*., chlorobenzene), anisole combines effective solvation properties with a more favorable safety and environmental profile, making it a greener alternative.^44^ While anisole itself is not edible and residual solvent content therefore remains a concern, the existence of structurally related anisole derivatives used in food-related applications makes it an attractive solvent model system.^45^

In contrast to films deposited from THF, β-carotene films processed from anisole exhibit clear signs of crystallinity already in the as-cast state. 2D-GIWAXS patterns (Figure 4c) show anisotropic (low angular distribution) diffraction peaks, representative of crystalline ordering with specific preferential orientations. The overall intensity is modest, owing to the reduced thickness of anisole-cast films (20 nm) when compared to THF (60 nm). Polarized optical microscopy (Figure 4a) reveals uniform birefringence consistent with a polycrystalline 2D-like texture. AFM topography (Figure 4b) further confirms the distinct morphology: the films exhibit extended 2D-domains with fibrillar substructures, markedly different from both the smooth amorphous texture of the as-cast films and the nanocrystalline surface of annealed crystalline THF-based films. These differences stem from the distinct thermodynamic and kinetic profiles governing solution crystallization in anisole versus THF. Crystallization from solution typically involves two main steps: nucleation, where molecular clusters form once supersaturation is reached, and growth, where these nuclei expand into ordered domains.^46^ Here, anisole’s lower solubility for β-carotene leads to faster supersaturation upon solvent evaporation, while its high boiling point slows the drying process, extending the window for nucleation and molecular reorganization. Moreover, the presence of preaggregated clusters may act as seeds for heterogeneous nucleation during deposition, further lowering the energy barrier for crystallization and guiding molecular ordering, similar to templated crystallization described in the literature.^47,48^ This crystallization route is accompanied by a lower nucleation rate, when compared to annealed THF-films, that allows crystal growth to dominate, resulting in the formation of large, ordered domains. Upon thermal annealing, the microstructure of anisole-cast films is essentially stable until approximately 80 °C, with only a slight increase in the terrace boundaries (Figures S12, S13). Above this annealing temperature, AFM reveals the emergence of a disordered, coarser texture disrupting the originally crystalline, continuous microstructure and leading to poor interconnectivity, similar to the case for THF-based films. Interestingly, DSC thermographs (Figure S14) exhibit an exothermic peak at around 106 °C, providing further evidence of how cold crystallization in this temperature range in β-carotene films results in the abrupt formation of coarse crystallites, when compared to the large, uniform domains driven by solution crystallization. Simultaneously, at temperatures above 80 °C, birefringent features observed under POM become progressively less defined, pointing to increased disorder (Figure S15). As a result, thermal annealing in this range degrades rather than improves the structural coherence and electronic performance of anisole-cast films. This evolution is directly reflected in the device performance. OFETs based on as-cast or mildly annealed anisole films (up to 60 °C) exhibit high field-effect mobilities, reaching values exceeding 10^–2^ cm^2^/(V s) (Figure 4d,e,f and Figure S16). This performance stems from the initially well-connected, uniform, polycrystalline 2D morphology that enables efficient charge percolation. However, as annealing progresses above 80 °C, and the texture coarsens, charge transport is increasingly hindered by disrupted connectivity and the formation of large isolated domains. Devices annealed at higher temperatures show a dramatic drop in mobility, up to 4 orders of magnitude, along with more pronounced nonidealities in the transfer and output characteristics, similar to THF-based devices (Figure S16).

**Figure 4:**
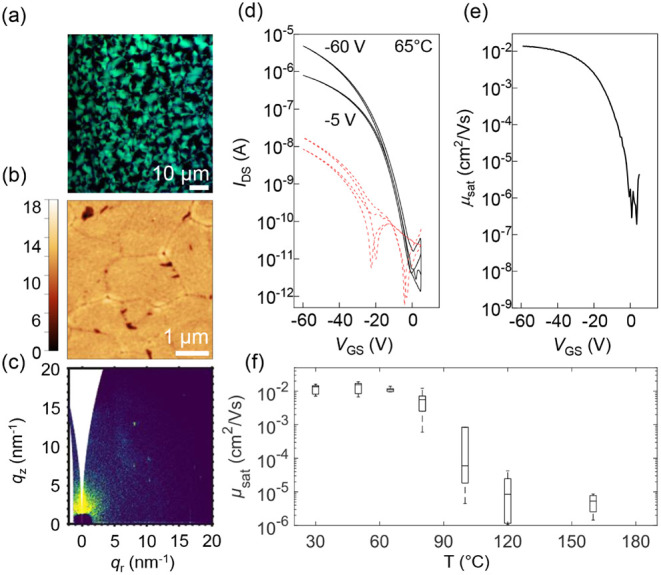
β-carotene cast from anisole. (a) Polarized optical micrograph, (b) AFM image, and (c) 2D-GIWAXS pattern of thin films as cast from anisole. (d) Representative transfer curve for TGBC transistors annealed at 65 °C. *V*_DS_ as indicated, gate current is shown in the red dashed line. Channel length *L* = 10 μm, and channel width *W* = 2 mm. (e) Corresponding charge carrier mobility in the saturation regime. (f) Charge carrier mobilities extracted in the saturation regime as a function of annealing temperature.

Overall, anisole-processed films yield well-performing OFETs, with charge carrier mobilities slightly higher than those obtained from THF-processed films and reduced data variability. These improvements can be attributed to the 2D morphology, which promotes more uniform charge percolation pathways and higher charge carrier mobility when compared to the irregular, small domain structure observed in THF-processed films.

To gain deeper insight into the molecular arrangement within the crystalline domains, we compared our experimental GIWAXS patterns with simulated diffraction maps based on the reported single-crystal structure of β-carotene (ICCDC deposition number: 1120466). We first focused on films processed from anisole. Considering that the same diffraction pattern is observed consistently across the annealing range from 30 to 120 °C (Figure S12), we then selected films annealed at 120 °C (Figure 5a), as they exhibit the highest diffraction intensities. We then simulated the 2D-GIWAXS pattern of the single-crystal structure assuming β-carotene molecules oriented along the (001) direction with respect to the substrate. The resulting *q*-map (Figure 5b) shows excellent agreement with the experimental data, with only a slight shift toward higher *q*-values, likely due to the unit cell relaxation in the film. We then analyzed β-carotene films processed from THF and annealed at 120 °C. In contrast to anisole-cast films, the corresponding 2D-GIWAXS patterns display a more complex diffraction signature, characterized by a combination of broad Bragg peaks and diffuse ring-like features. To assess whether the molecular packing retains any resemblance to the known single-crystal structure, we compared the azimuthally integrated GIWAXS profile with a simulated powder XRD pattern derived from the single-crystal data (Figure S17). However, this comparison showed poor agreement, indicating that the crystal packing in the THF films significantly deviates from that of the bulk single crystal.

**Figure 5:**
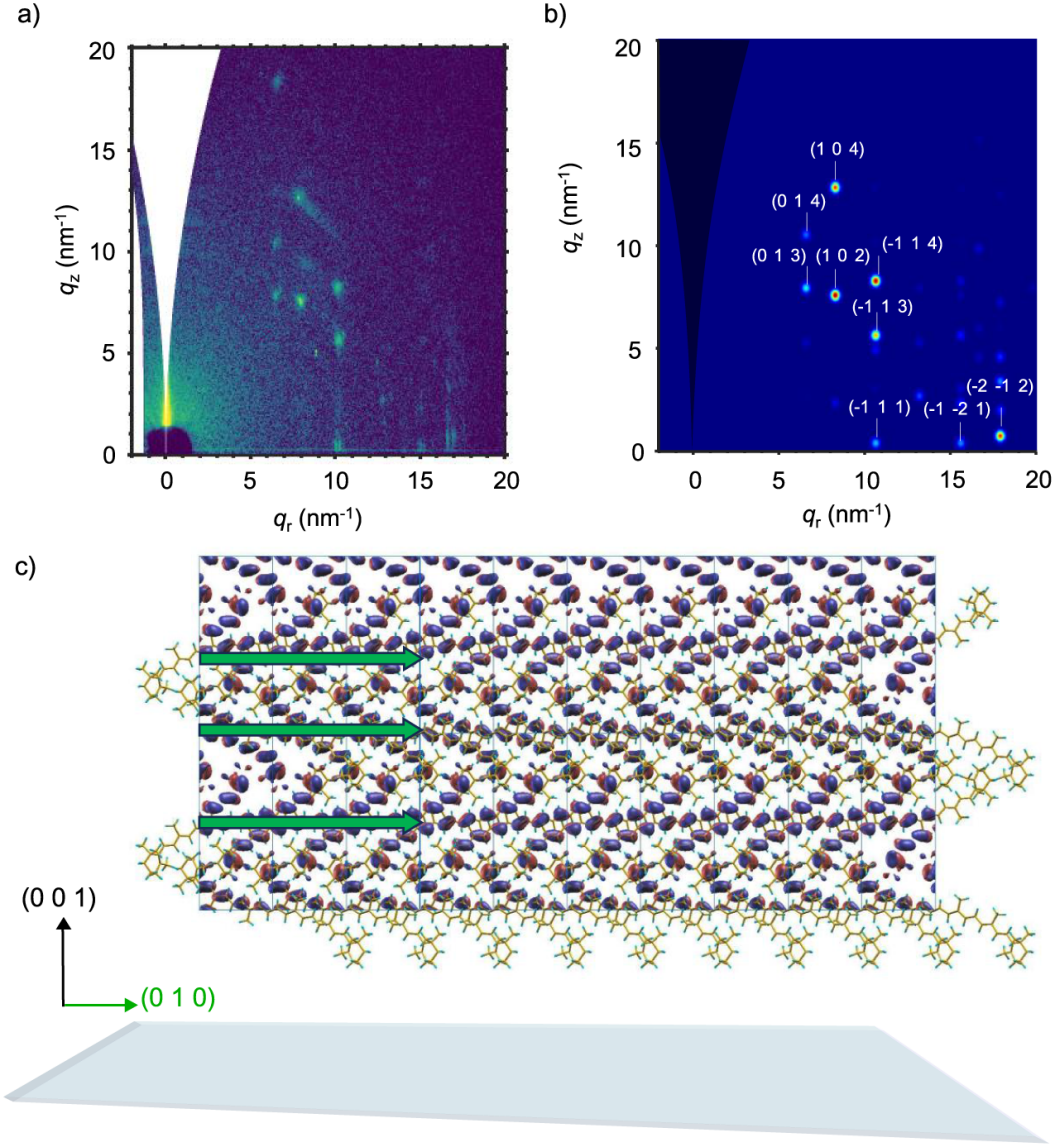
(a) 2D-GIWAXS pattern of β-carotene films processed from anisole and annealed at 120 °C for 5 min. (b) Simulated 2D-GIWAXS film diffraction pattern (ICCDC deposition number: 1120466) of β-carotene molecules oriented along the (0 0 1) direction with respect to the substrate, as visible from the sketch in (c), which also shows the DFT-computed highest occupied molecular orbital (HOMO) for the dimer and the (0 1 0) direction (green arrows).

Given the poor initial fit, we then carried out a Le Bail refinement of the azimuthally integrated diffraction pattern, allowing for shifts in the unit cell parameters to account for lattice relaxation, thermal expansion, and solvent effects. The refinement yielded a substantially improved fit, suggesting that the refined unit cell better captured the average crystallographic features present in the film (Figure S18). However, simulating a 2D-GIWAXS pattern based on the refined structure proved challenging due to the complexity of the texture, the likely presence of multiple orientations, and possible contributions from unknown polymorphs. Nevertheless, a qualitative comparison using simulated *q*-maps of crystallites oriented along the (0 1 −3) and (0 0 2) directions provided partial overlap with features in the experimental pattern (Figure S18), hinting at the coexistence of different crystal families with specific preferential orientations. This complex microstructure may stem from the heterogeneous energetic environment in which crystallization occurs in thermally annealed THF-cast films: an amorphous film constrained between the substrate interface and the ambient atmosphere, which could lead to the growth of two distinct crystal families with different orientations. In contrast, solution-phase crystallization from anisole takes place during solvent evaporation in a more homogeneous energetic landscape, where enhanced molecular mobility facilitates the formation of a uniform crystal phase, resembling the growth conditions reported to achieve single crystals.

To complement the structural insights obtained from GIWAXS simulations, we carried out density functional theory (DFT) calculations to assess the intrinsic charge transport parameters of β-carotene. The analysis was performed on a single-crystal structure. By leveraging the crystallographic information, we were able to extract key quantities such as the intramolecular reorganization energy and the transfer integrals along defined crystallographic directions, and compare the results with those obtained in the case of a selection of well-investigated organic semiconductors (see Tables S2–S3 for a complete account), providing a microscopic basis for understanding the charge transport. The calculations yielded an intramolecular reorganization energy (λ^+^) of 0.31 eV (B3LYP) and 0.51 eV (M06-2X), values that fall within the expected range for organic semiconductors, though at the higher end. To assess electronic coupling, transfer integrals (*J*) were computed along key crystallographic directions using the energy splitting in dimers (ESD) approach within Koopmans’ approximation. The largest transfer integral was found along the (0 1 0) direction, with *J* values of 0.05 eV (B3LYP) and 0.064 eV (M06-2X). Figure S19 shows the strong similarities between periodic and dimer HOMO orbitals of β-carotene along the (0 1 0) direction. In contrast, the (1 0 0) and (1 1 0) directions yielded *J* values of only ∼0.002 eV, highlighting the strong anisotropy of charge transport in β-carotene. These values are comparable with those calculated in the case of pentacene, rubrene, and dioctyl[1]­benzothieno­[3,2-*b*]­[1]­benzothiophene using the same methods, as summarized in Tables S2 and S3. To visualize how this pronounced 1D character translates into the crystalline domains of β-carotene films, Figure 5c shows the (0 1 0) direction together with the molecular arrangement with respect to the substrate in the case of anisole-cast films. Clearly, this molecular arrangement is particularly favorable for the charge transport in coplanar OFETs, as the (0 1 0) direction, which offers the highest transfer integral, lies parallel to the substrate (Figure 5c and Figure S19). This strong anisotropy also implies that charge transport is highly sensitive to the orientation and connectivity of the crystalline domains. As a result, films with a high density of well-defined grain boundaries, as seen in all the THF-cast films, especially the overannealed ones, tend to exhibit lower mobility, despite their overall crystallinity. This reinforces the importance of processing conditions that promote ordered film growth, such as the use of anisole, which favors crystalline domains with directional connectivity aligned with the most conductive axis.

Having established that charge transport of β-carotene can be tailored by controlling its structural properties and molecular arrangement, we next investigated whether this correlation extends to the material stability. Given the well-documented susceptibility of carotenoids to environmental degradation, particularly under exposure to light, oxygen, and heat, their stability represents a critical limitation for their use in organic electronics. We therefore assessed how different processing conditions, leading to distinct microstructures, affect the stability of β-carotene thin films and OFETs under operational and ambient conditions. To simplify the analysis and enable a clearer correlation with microstructural evolution and crystallinity, we focused on devices processed from THF, which exhibit a well-defined transition from an amorphous to crystalline morphology upon annealing.

To evaluate the photostability of β-carotene thin films, we monitored the evolution of their UV–vis absorbance spectra under continuous illumination in air over time (Figure 6, bottom panel). Films annealed at 30 °C, corresponding to an amorphous microstructure, exhibited a significant decrease in absorbance and a complete loss of vibronic structure, indicating extensive photoisomerization and/or oxidative degradation.^49,50^ In contrast, films annealed at 60 and 120 °C, both exhibiting progressively higher degrees of crystallinity, displayed markedly improved spectral stability, with the highest crystallinity film showing only minor decreases in peak intensity and no significant spectral shifts. These results suggest that increased molecular ordering can hinder conformational flexibility and reduce exposure to reactive species, thereby enhancing resistance to photodegradation, even in intrinsically unstable films.

**Figure 6:**
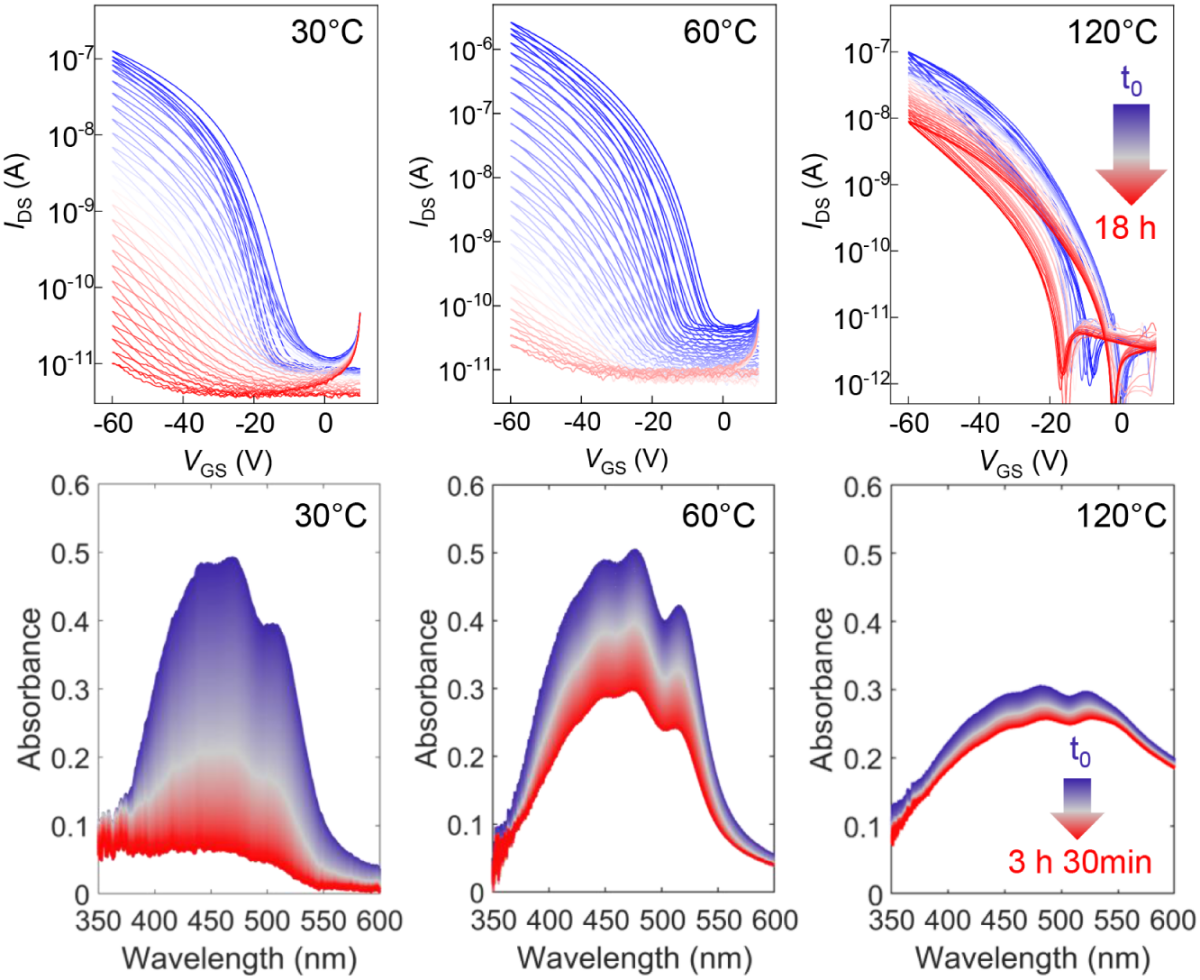
Stability of β-carotene cast from THF, annealing temperature as indicated. (top) Transfer characteristics measured in air as a function of time. Devices were measured every 30 min for 18 h. (bottom) UV–vis absorption spectra as a function of time in air upon illumination.

To correlate these findings with device performance, we tracked the transfer characteristics of OFETs over 18 h of storage in ambient air (in the dark; Figure 6, top panel). The 18-h time window was selected to enable a consistent comparison across all processing conditions, as both disordered and partially ordered devices undergo complete electrical degradation within this time frame. While this observation period is sufficient to capture relative stability trends, it does not meet the shelf-life requirements of practical devices. In real-world edible electronic systems, the semiconductor layer would not operate in isolation but would be integrated within a multilayer edible stack, where encapsulation layers are expected to dominate the operational lifetime by limiting exposure to oxygen and moisture. Consequently, the stability data presented here should be interpreted as an assessment of the *intrinsic* stability of β-carotene films and devices rather than a prediction of the lifetime of fully engineered, encapsulated edible electronics.

Devices based on disordered (30 °C) and partially ordered (60 °C) films rapidly lose channel modulation. A similar degradation kinetics is also observed in anisole-cast devices (Figures S20, S21). In contrast, OFETs based on the more crystalline films (120 °C) showed the best resilience with only a moderate decrease in current (hence mobility) throughout the test (Figure S21). Thus, relatively thick (≈100 nm, Figures S7, S8), well-ordered, faceted 3D-crystallites lead to an increased electronic stability, despite not providing the most convenient texture for OFET architectures. The other films, comprising either a larger amorphous fraction (as-cast/mildly annealed THF films) or thin 2D platelets with a certain degree of pinholes/defects (anisole films), are not able to screen sufficiently the degradation processes. Effectively, these trends confirm that the structural arrangement of β-carotene not only governs its charge transport properties but also plays a key role in stabilizing the material under operationally relevant conditions.

## Conclusions

We have shown how β-carotene, a naturally available edible compound, can serve as a viable semiconducting material for organic electronic devices. Notably, the charge-transport properties demonstrated here place β-carotene in the same performance range as established organic semiconductors such as CuPc and P3HT, which have been explored within broader efforts toward edible or biocompatible electronics. While CuPc offers superior environmental stability, its processing typically relies on more complex or specialized deposition approaches. In contrast, polymer semiconductors such as P3HT are not qualified as edible materials. In this context, β-carotene emerges as a unique example of a food-derived semiconductor combining competitive transport properties with straightforward solution processing.

Through the investigation of processing–structure–property relationship, we show how solvent selection and thermal treatment dramatically influence molecular organization, morphology, and charge transport properties of β-carotene. While THF-cast films require postdeposition annealing to induce crystallinity and achieve improved device performance, anisole-processed films exhibit an intrinsically ordered microstructure in the as-cast state, yielding high and consistent field-effect mobilities exceeding 10^–2^ cm^2^/(V s). Our results are further supported by structural simulations and electronic structure calculations, confirming the correlation between molecular packing and transport efficiency. Importantly, we show that microstructural control also plays a critical role in enhancing the environmental stability. Films made of highly crystalline domains, despite not showing the best transport properties in OFETs, exhibit significantly greater resistance to photodegradation and electrical performance loss under ambient conditions compared to their more disordered and defective counterparts, demonstrating that structural ordering can mitigate one of the major limitations of carotenoid-based materials.

These insights into structure–property relationships lay the foundation for future electronic devices that combine high performance with enhanced stability, providing a framework for integrating β-carotene into fully edible electronic stacks. To do so, incorporation with edible substrates, electrolytes, contacts, and gate dielectrics, which have already been reported by our group and others, is clearly the next step. Here the challenges primarily concern solvent orthogonality during multilayer fabrication, requiring an engineering optimization pathway and the implementation of low-voltage operation through suitable edible electrolytes. In this context, assessing the stability of carotenoid-based semiconducting films when interfaced with aqueous or electrolyte-rich environments represents an important aspect to be addressed in future studies.

Moreover, beyond demonstrating the potential of β-carotene as an edible semiconductor, this study highlights how the methodological tools developed in the field of organic electronics can be leveraged to unlock the full potential of naturally derived materials, providing a foundation for the rational design of a new class of environmentally conscious electronic devices. Yet, achieving this scenario critically depends on the development of cost-effective and efficient extraction methods capable of isolating individual carotenoid species, making biosourced semiconductors truly sustainable. While the extraction of carotenoid mixtures from natural sources is well established, the selective and sustainable isolation of single compounds remains a key technological challenge.^51,52^ Addressing this limitation will be essential to fully realize the promise of edible and biodegradable electronics and represents an important direction for future research.

## Methods

### Materials

Tetrahydrofuran (THF) ≥99.9% anhydrous, Anisole ≥99.7%, Poly­[4,5-difluoro-2,2-bis­(trifluoromethyl)-1,3-dioxole-*co*-tetrafluoroethylene] (PTFE/Teflon) dioxole 65 mol %, Fluorinert FC-40, β-carotene synthetic with purity >93% (UV), were acquired from Merck and used as received. Gold, Chromium, and Aluminum were acquired from Fisher Scientific. Glass substrates (low alkali 1737F Corning glasses) were purchased from Präzisions Glas & Optik GmbH.

### Sample Preparation

β-Carotene was dissolved in THF or anisole at a concentration of 5 mg mL^–1^. The obtained solutions were stirred at 60 °C for 30 min in N_2_ atmosphere. The anisole-based solution was then filtered through a PVDF 0.2 μm filter to eliminate precipitates. The two types of solutions were spin-cast onto either glass substrates or silicon substrates in a N_2_ atmosphere at a speed of 1000 rpm for 1 min and then thermally annealed at the above-mentioned temperatures on a previously heated hot plate for 5 min. The substrates were cleaned in an ultrasonic bath before use, in acetone for 5 min, and subsequently in isopropyl alcohol for 5 min. Afterward, the substrates were cleaned in an O_2_ plasma for 5 min to remove eventual organic residues and to improve wettability. To avoid inhomogeneities due to the limited thickness of the substrates, a sacrificial microscope glass slide was placed between the spin coater chuck and the substrates during the spin-coating step.

### Organic Field-Effect Transistors

β-Carotene-based OFETs were fabricated with a bottom-contact, top-gate configuration onto 2 × 2 cm^2^ glass substrates. The interdigitated source and drain electrodes of Cr/Au (3/30 nm) were deposited via photolithography. A positive photoresist, AZ5214E, was spin-coated onto the substrate at 6000 rpm for 60 s. The resist was then annealed at 110 °C for 90 s. The chosen pattern was written with a 365 nm UV light into the resist to locally cross-link it with a maskless aligner (Heidelberg MLA100). Afterward, the resist underwent a development step with a metal ion-free solvent, MIF726, for 25 s. To grant good adhesion between the glass substrate and the contacts, the metal stack chosen for the electrodes comprised 3 nm of Chromium and 30 nm of Gold, which were thermally evaporated one on top of the other without breaking the high vacuum in the chamber. Lastly, the substrates were immersed in Technistrip 2 for the liftoff process. The as-obtained substrates were cleaned following the procedure reported in the previous paragraph. β-Carotene thin films were deposited following the procedure reported in the previous paragraph. The Teflon (60 g L^–1^) dielectric layer was spin-coated in two steps: 1000 rpm for 60 s and then 3000 rpm for 60 s, and subsequently annealed at 35 °C for 30 min, obtaining a final thickness of approximately 600 nm. Finally, the top gate electrode (Al) was deposited via thermal evaporation through a shadow mask in a Moorfield thermal evaporator.

The electrical characterization was generally performed in a N_2_ atmosphere using an Agilent B2902A semiconductor parameter analyzer. The stability measurements were conducted with the same setup but in ambient air. For each device type and processing condition, electrical measurements were performed on at least 15 devices, obtained from multiple fabrication batches.

### UV–Vis Spectroscopy

The absorption spectra as a function of the annealing temperature for thin films on glass substrates were acquired with a PerkinElmer Lambda 1050 UV/vis/NIR spectrometer by measuring transmission spectra. The UV–vis absorption spectrum of the isolated molecule was measured in a dilute solution with a concentration of 1 mg L^–1^ using a quartz cuvette. For the stability measurements, absorption spectra were recorded by transmitting visible light from a halogen lamp (High Power UV–vis Fiber Light Source, Hamamatsu Photonics) through the thin film and detecting it with a fiber-coupled Ocean Optics Maya Pro 2000 spectrometer. The beam was first focused to a 1 mm spot and then collimated and refocused for detection. The power of the beam was 50 μW, and each spectrum was acquired with an integration time of 10 ms and a number of averages of 100.

### Optical Microscopy

Nonpolarized and Polarized optical microscopy images were acquired with a Zeiss Axio Scope A1 equipped with a single polarizer (EpiPol mode) using the reflection mode.

### Grazing Incidence Wide Angle X-Ray Scattering (GIWAXS)

GIWAXS measurements were performed at the BL11-NCD-Sweet beamline at the ALBA Synchrotron Radiation Facility in Barcelona (Spain) with a Rayonix WAXS LX255-HS detector. The incident energy was 12.4 eV, and the sample-to-detector distance was 194.489 mm. The angle of incidence ranged between 0.1° and 0.12°, while the exposure time ranged between 1 and 5 s. 2D-GIWAXS patterns were corrected as a function of the components of the scattering vector with a Matlab script developed by Aurora Nogales and Edgar Gutiérrez (https://mathworks.com/matlabcentral/fileexchange/71958-grazing-incidence-wide-angle-x-ray-scattering-representation).^53^ Thin films were fabricated following the same process and route described above onto highly doped silicon substrates.

### GIWAXS Pattern Simulation

2D-GIWAXS simulated diffraction patterns were obtained by SimDiffraction.^54^ The simulations were initialized by choosing a specific molecular orientation (Miller index) from the related .CIF file (CCDC deposition number: 1120466)^55^ with respect to the substrate. The Miller indices (h k l) were previously determined by Mercury,^56^ a 3D crystal structure visualization and exploration software. Mercury was also used to obtain the simulated powder X-ray diffraction patterns for a given single crystal structure. GSAS-II^57^ was used to perform Le-Bail refinement of the total azimuthal integrated β-carotene *q*-maps.

### Atomic Force Microscopy (AFM)

AFM characterization was performed with an Agilent 5500 atomic force microscope operating in the acoustic mode. Surface topography images were processed by using Gwyddion image analysis software.

### Differential Scanning Calorimetry (DSC)

DSC measurements were performed with a DSC 1 STARe System from Mettler Toledo. β-Carotene solutions are drop-cast on glass slides. The film is then scratched from the substrates, and the material is loaded into aluminum crucibles. Measurements were conducted under N_2_ flow (80 mL min^–1^) with heating/cooling rates of 10 °C/min.

### Density Functional Theory (DFT) Calculations

(Time-dependent) DFT simulations were performed using a two-step protocol. Crystal structures have been investigated using periodic calculations in a plane-wave/pseudopotential framework, as implemented in the Quantum ESPRESSO suite of programs.^58,59^ Reorganization energies, transfer integrals, and absorption spectra have been then calculated on isolated molecules and dimers extracted from optimized periodic structures in an all-electron/localized-basis-set framework, using the ORCA suite of programs.^60,61^ A complete account of theoretical methods is reported in the Supporting Information.

## Supplementary Material



## Notes

Additional experimental details and characterization data for β-carotene films, including OFET transfer and output characteristics, UV–vis absorption, AFM, optical microscopy, DSC, and GIWAXS analyses; DFT and TD-DFT computational methods and results (PDF)

## □

nanotech@surfaces Laboratory, Empa – Swiss Federal Laboratories for Materials Science and Technology, Dübendorf, 8600, Switzerland
