# Supplementary material for: Food-Based Electronics: Revisiting β‑Carotene Organic Transistors
Source: ACS Appl Mater Interfaces. 2026 Feb 23;18(9):14056–66. doi: 10.1021/acsami.5c23614 (PMC12983197; doi:10.1021/acsami.5c23614)
Supplement: Supplementary file 1 [file am5c23614_si_001.pdf]

**Supporting Information**

# Food-based electronics: revisiting β-carotene organic transistors

*Alberto D. Scaccabarozzi,*[a b † *] *Elena Feltri,*[a †] *Pierluigi Mondelli,*[a] *Pietro Rossi,*[a] *Francesca Pallini,*[c] *Antonella Treglia,*[a] *Annamaria Petrozza,*[a] *Luca Beverina,*[c] *Giuseppe Mattioli,*[d] *Jaime Martin,*[e] *Alessandro Luzio* [a *] *and Mario Caironi* [a *]

a. Center for Nano Science and Technology, Istituto Italiano di Tecnologia, Via Rubattino, 81, Milano, 20134 Italy.

b. Department of Physics, Politecnico di Milano, Piazza Leonardo da Vinci, 32, Milano, 20133 Italy.

c. Department of Materials Science, University of Milano-Bicocca, Via R. Cozzi, 55, Milano, 20126 Italy.

d. Consiglio Nazionale delle Ricerche (CNR), Istituto di Struttura della Materia (ISM), Strada Provinciale, 35d/9, 00010 Montelibretti, Italy.

e. Universidade da Coruña, Campus Industrial de Ferrol, CITENI, 15403, Esteiro, Ferrol, Spain.

† Alberto D. Scaccabarozzi and Elena Feltri equally contributed to the work.

Corresponding author's email address:

alberto.scaccabarozzi@polimi.it

alessandro.luzio@iit.it

mario.caironi@iit.it

**Figure S1-S10:** THF-based system

**Figure S1-S3**: Representative transfer and output characteristics, along with charge-carrier mobility extracted in the saturation regime, for Organic Field-Effect Transistors (OFETs) based on β-carotene films processed from THF. As cast films show low performance in the as-cast state, with mobility values in the range of $10^{-4}$ $cm^2/Vs$. Upon annealing, a significant enhancement in charge transport is observed progressively up to 60°C, with mobilities reaching $10^{-2}$ $cm^2/Vs$ for the best devices. This improvement is attributed to thermally induced crystallization. However, further annealing above this temperature leads to performance degradation due to morphological coarsening and reduced connectivity. All devices have a channel length ($L$) of 10 µm, except those annealed at 90 °C. In this case, functional devices could only be obtained for shorter channel lengths ($L$ = 2.5 and 5 µm), as no working transistors were observed for $L \geq 10$ µm. For the 60 °C annealed films, both the best-performing and average devices are reported.

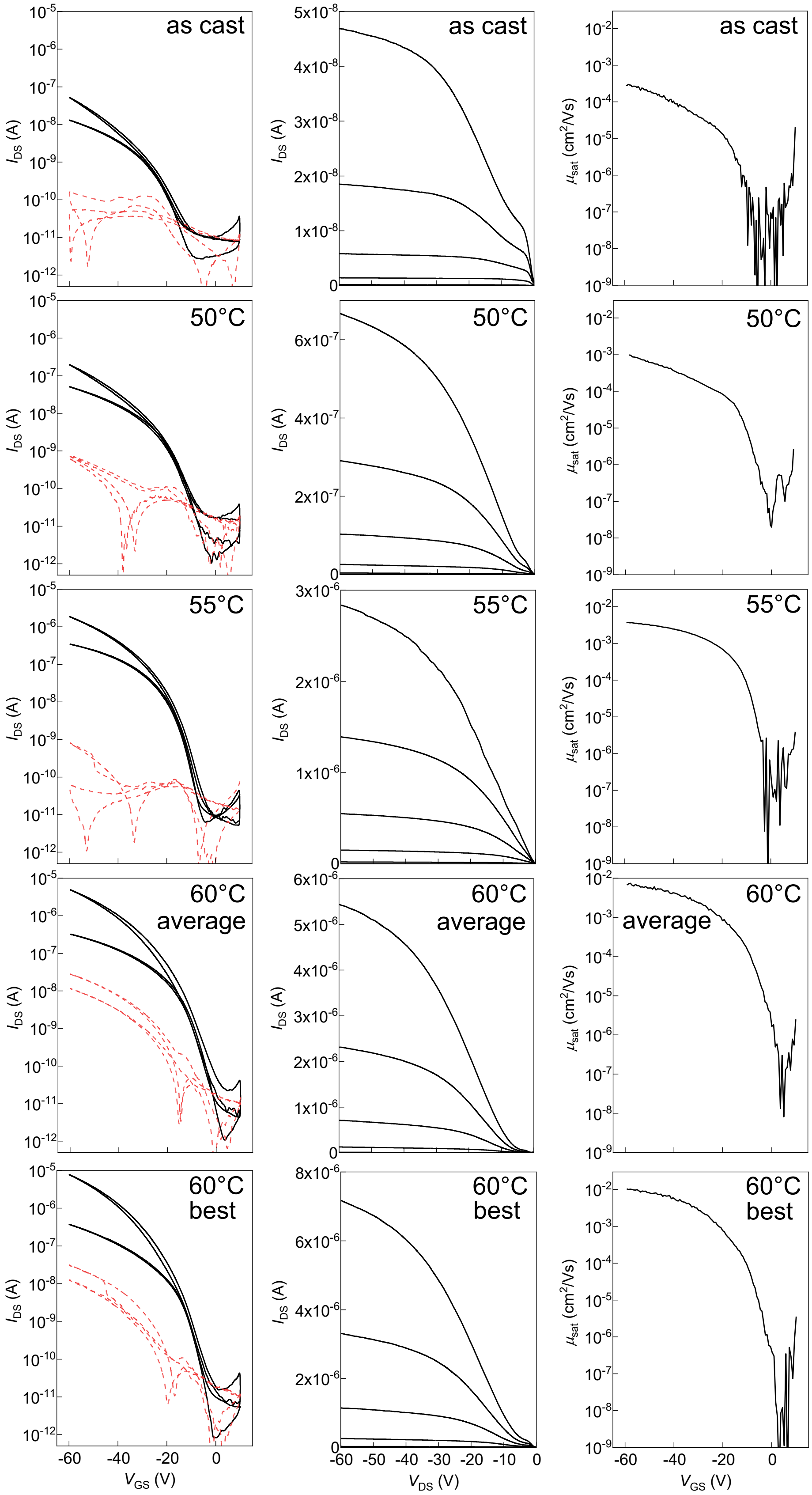


Figure S1: TGBC transistors processed from THF, annealed as indicated. (left) Representative transfer curves measured at $V_{DS}$ = -5 V and -60 V, gate current is shown in red dashed line. (center) Corresponding output characteristics with a $V_{GS}$ ranging from 0 V to -60 V, with a ΔV = -10 V and (right) charge carrier mobility in the saturation regime.

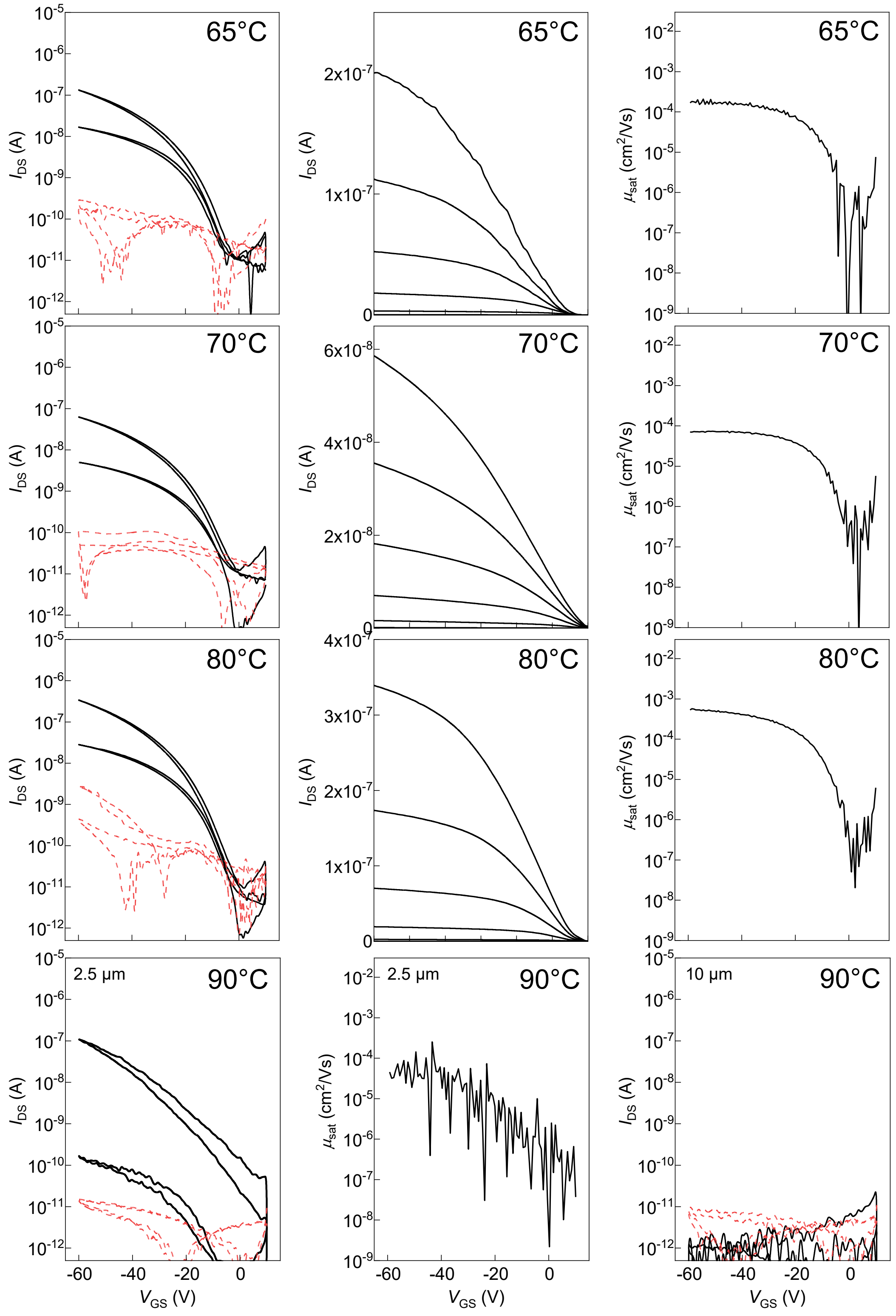


Figure S2: TGBC transistors processed from THF, annealed as indicated. (left) Representative transfer curves measured at $V_{DS}$ = -5 V and -60V, gate current is shown in red dashed line. (center) Corresponding output characteristics with a $V_{GS}$ ranging from 0 V to -60 V, with a ΔV = -10 V and (right) charge carrier mobility in the saturation regime.

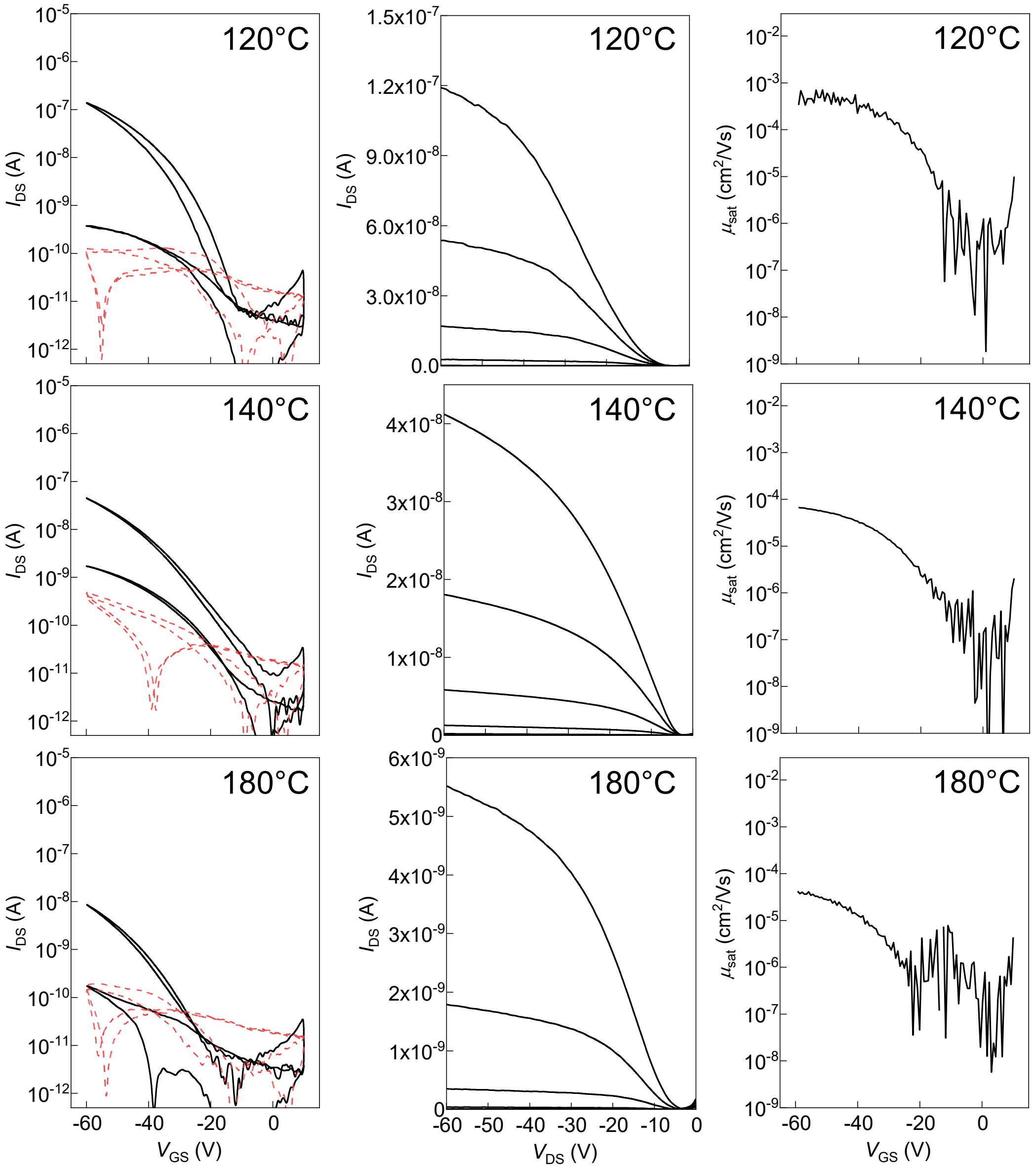


Figure S3: TGBC transistors processed from THF, annealed as indicated. (left) Representative transfer curves measured at $V_{DS}$ = -5 V and -60V, gate current is shown in red dashed line. (center) Corresponding output characteristics with a $V_{GS}$ ranging from 0 V to -60 V, with a ΔV = -10 V and (right) charge carrier mobility in the saturation regime.

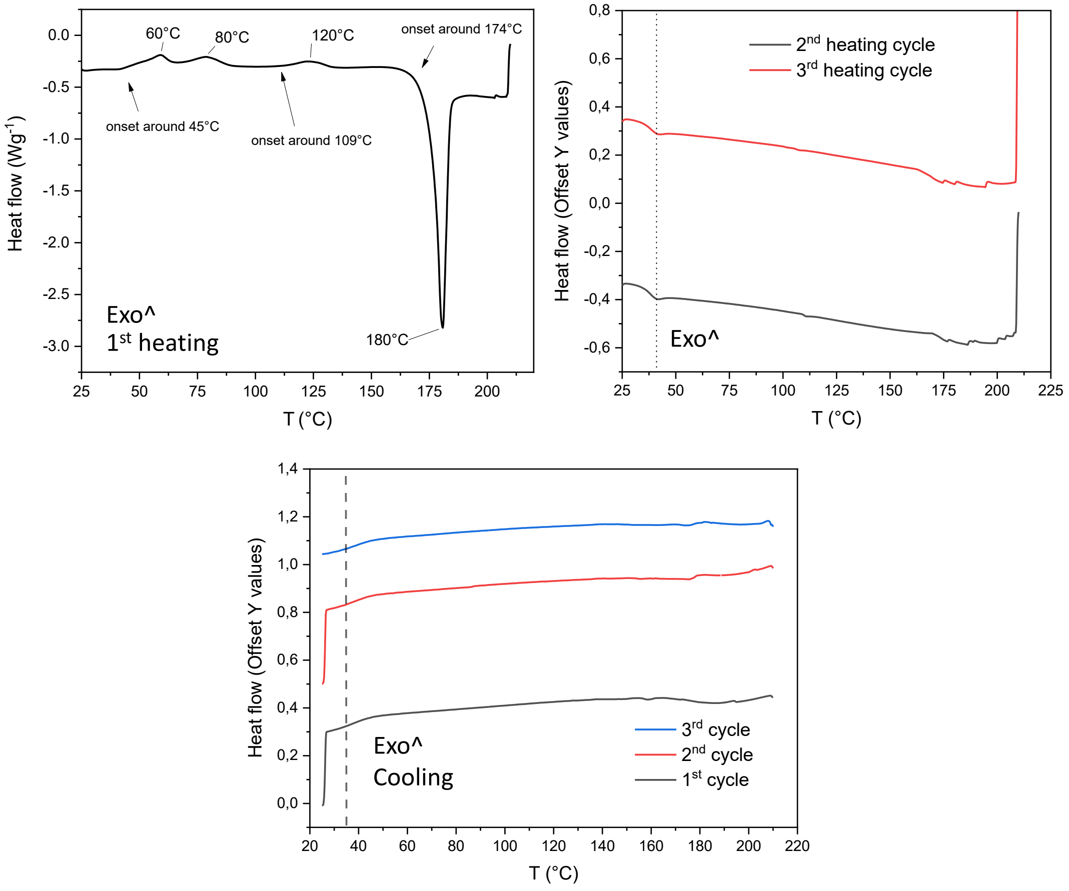


Figure S4: Differential Scanning Calorimetry (DSC) thermographs of THF-processed β-carotene.

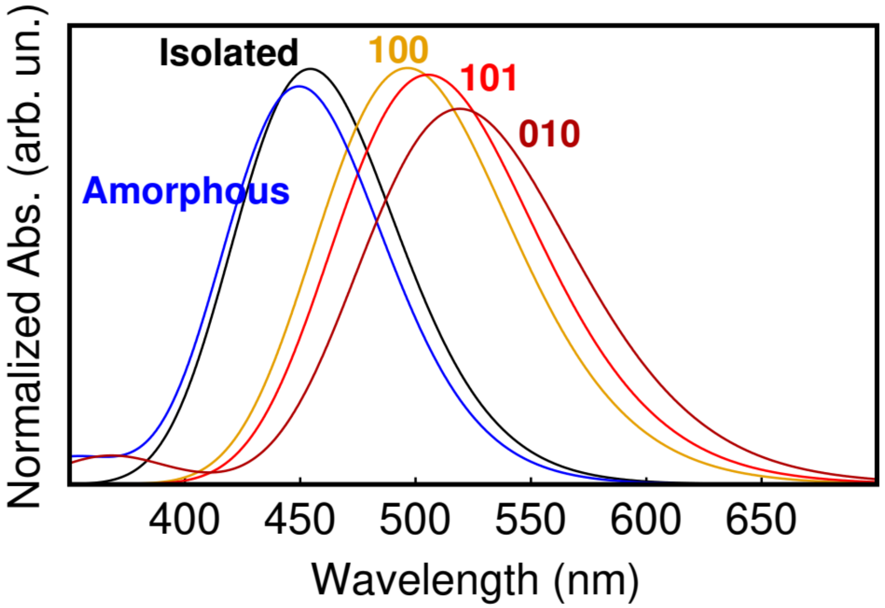


Figure S5: TDDFT absorption spectra of the isolated β-carotene molecule (black) and of a series of β-carotene dimers, extracted from the crystal structure along the indicated directions (orange to dark red) and mimicking the features of an amorphous film (blue). The spectra have been obtained as convolution of electronic transition using Gaussian Functions (FWHM = 4000 $cm^{-1}$).

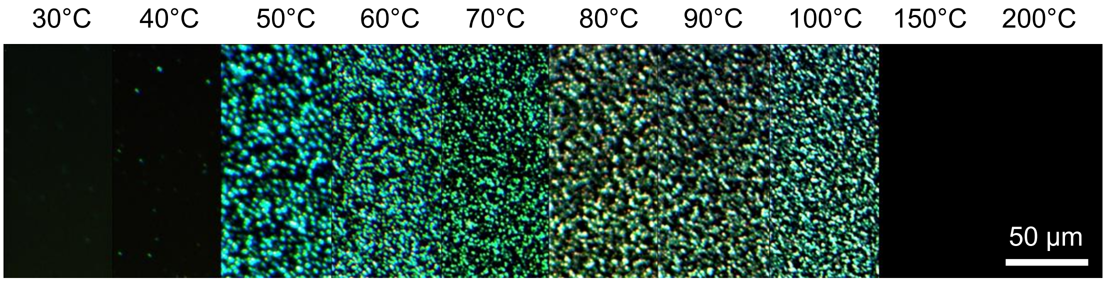


Figure S6: Optical micrographs under polarized light of THF-processed β-carotene films as a function of annealing temperature.

**Figure 7-8**: Atomic force microscopy (AFM) images of β-carotene films cast from THF upon annealing. In the as-cast state, films display a smooth and featureless topography, consistent with the amorphous structure observed in GIWAXS. Upon mild annealing at 60 °C, the surface becomes more heterogeneous, with the appearance of dispersed, thick aggregates—some reaching up to ≈100 nm in height—embedded within a continuous matrix. As the annealing temperature increases, the texture becomes coarser, evolving into well-defined, faceted structures that resemble highly ordered crystals. The coarsening of these domains is accompanied by the emergence of grain boundaries and surface roughening, which disrupt the continuity of the film and correlate with the decline in device performance observed at higher annealing temperatures.

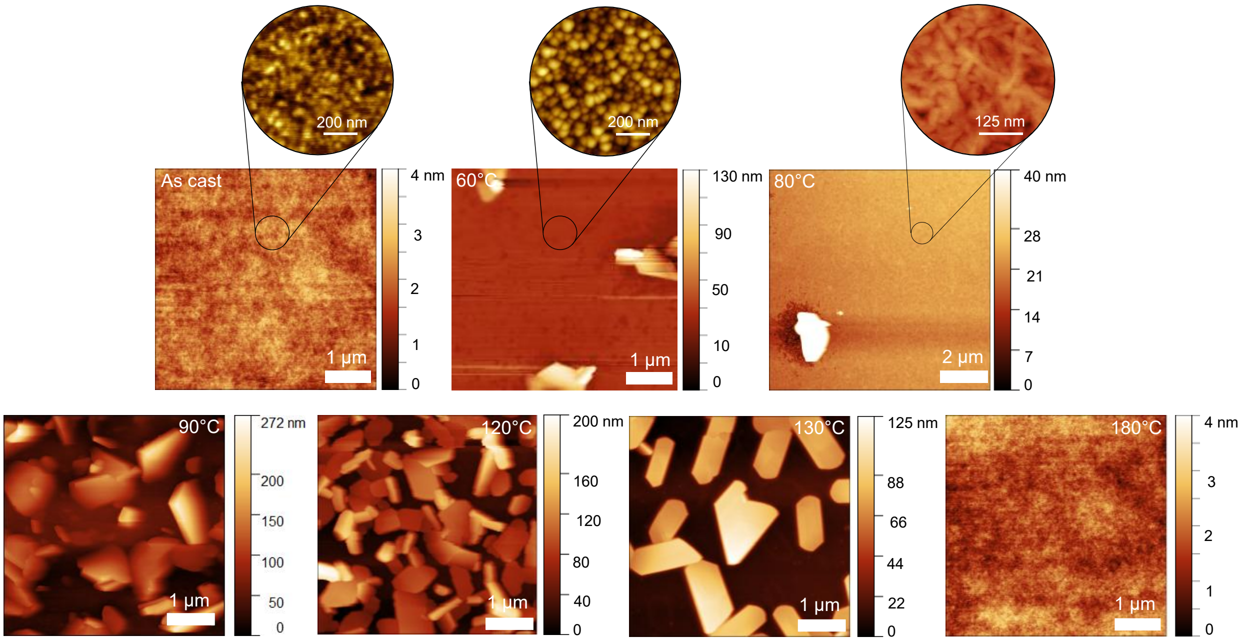


Figure S7: AFM images of β-carotene thin-films cast from THF annealed at temperatures as indicated.

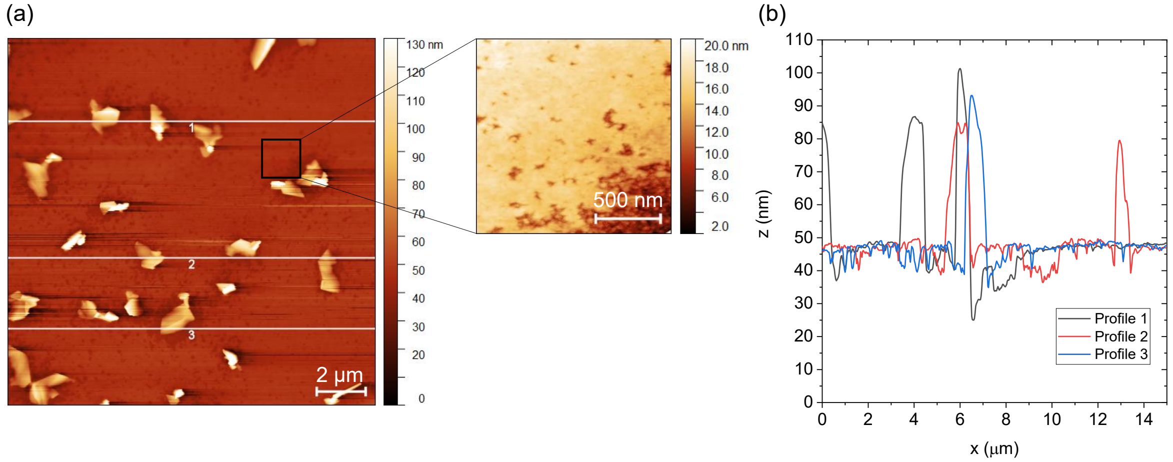


Figure S8: (a) AFM image of β-carotene thin-films cast from THF annealed at 60°C. The onset shows an area of the film next to a big aggregate. (b) Height profiles of three profiles, as indicated.

**Figure S9-S10**: 2D-GIWAXS and corresponding profiles along the in-plane and out-of-plane directions for THF-processed β-carotene films.

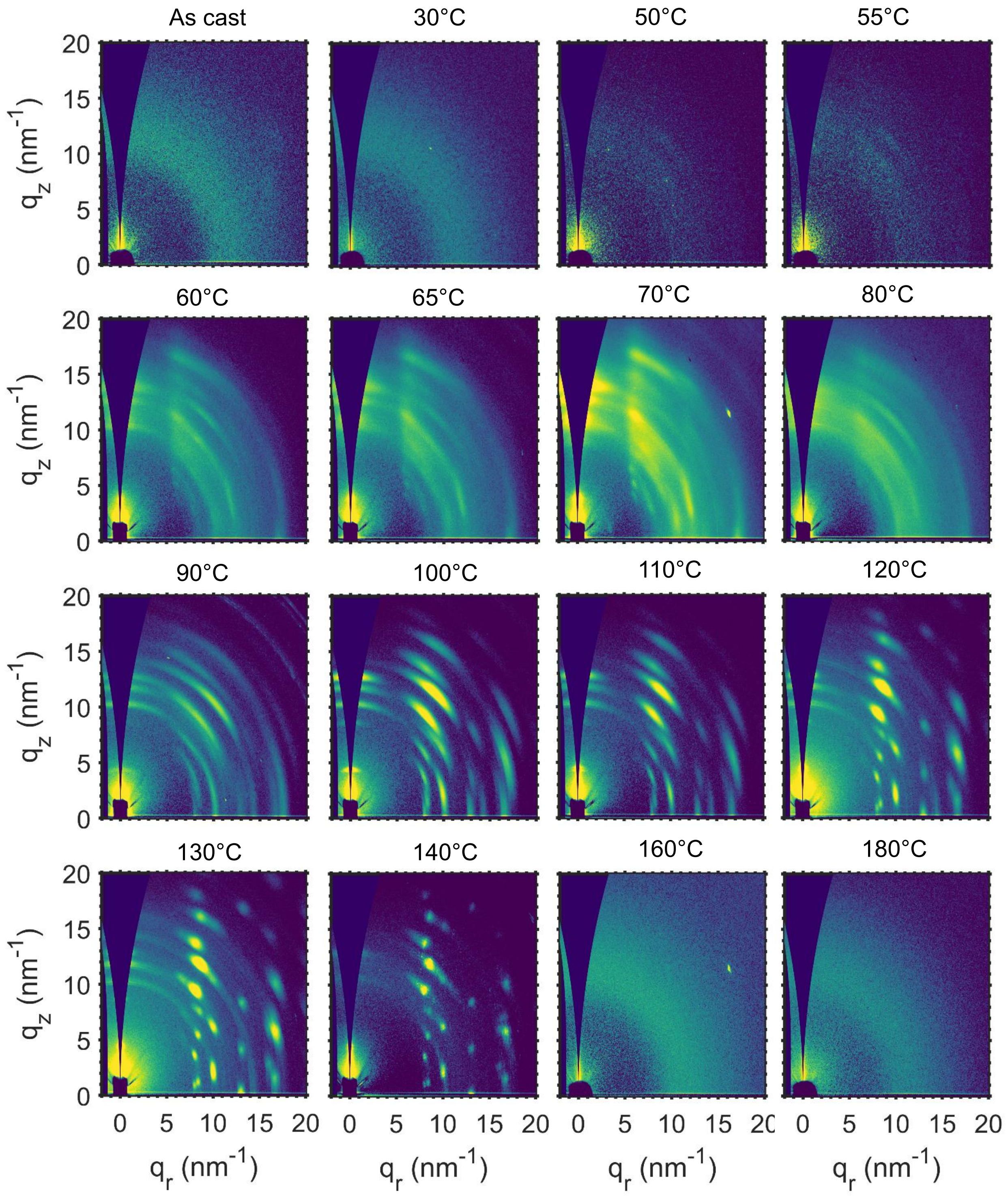


Figure S9: 2D-GIWAXS patterns of films cast from THF, annealed in glovebox for 5 min at the temperature indicated.

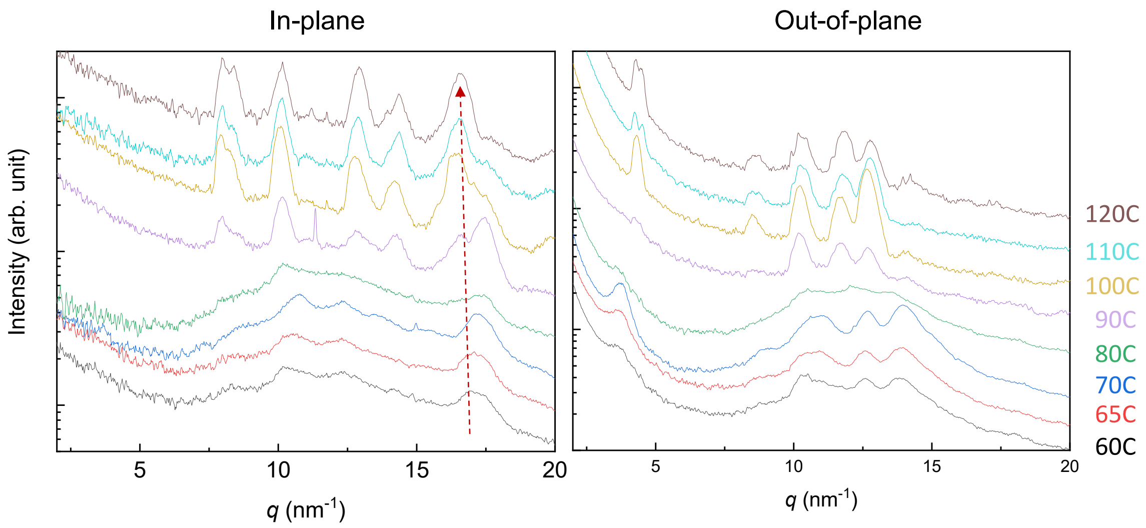


Figure S10: GIWAXS profiles along the in-plane and out-of-plane directions. Different annealing temperatures are indicated.

The crystalline coherence length ($L$) was estimated using a Scherrer-type analysis, according to equation 1:

$$L = 2\pi/\Delta q \quad (1)$$

Where $\Delta q$ is the full width at half maximum (FWHM) of selected GIWAXS reflections.

We focused the analysis on a specific diffraction peak at $q \approx 16.7$ nm$^{-1}$ (indicated with a red arrow in Figure S10), whose scattering vector is predominantly aligned with the π-π stacking direction, which plays a dominant role in charge transport.

**Table S1.** Full width at half maximum (FWHM) and crystalline coherence length ($L$) of diffraction peak at $q \approx 16.7$ nm$^{-1}$ as a function of annealing temperature.

| *Annealing Temperature* | *FWHM* | *L* | |
|---|---|---|---|
| 120°C | 0.69 | 9.11 | nm |
| 110°C | 0.73 | 8.61 | nm |
| 100°C | 0.73 | 8.61 | nm |
| 90°C | 0.7 | 8.98 | nm |
| 70°C | 0.75 | 8.38 | nm |
| 65°C | 0.75 | 8.38 | nm |
| 60°C | 0.7 | 8.98 | nm |

The extracted coherence length remains essentially unchanged across the annealing conditions investigated, indicating that thermal treatment primarily affects the amount and texture of crystalline material rather than the extent of coherent crystalline order. We note that the GIWAXS patterns exhibit multiple diffraction peaks that are

partially overlapping and difficult to unambiguously index, which limits the feasibility of a more comprehensive line-shape analysis.

**Figure S11-S16**: Anisole-based system

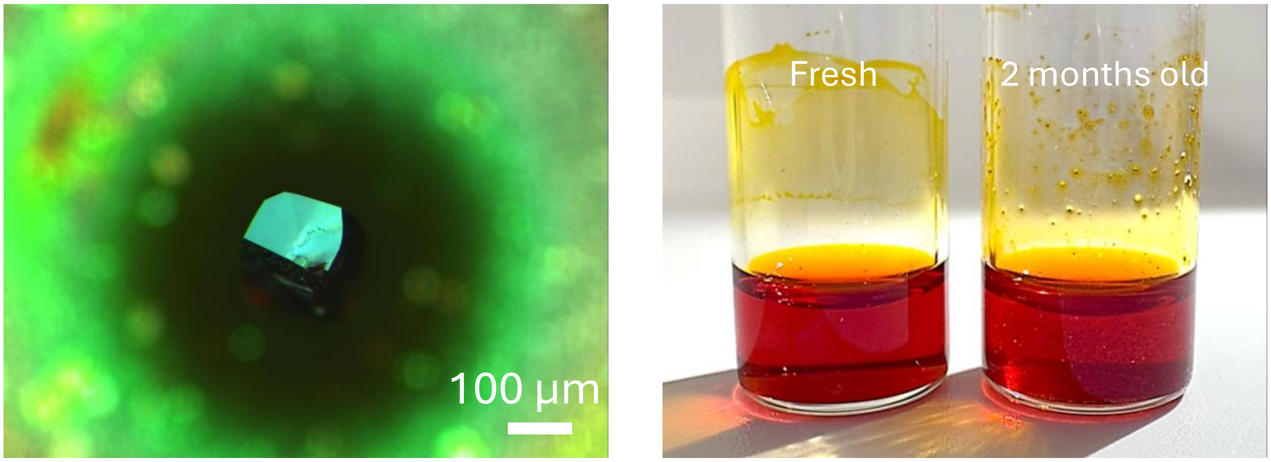


Figure S11: β-carotene crystal grown in anisole solution and cast on a glass slide (left). Fresh, filtered solution is clear, while progressive aggregation occurs within hours leading to crystallites visible at the naked eye (right).

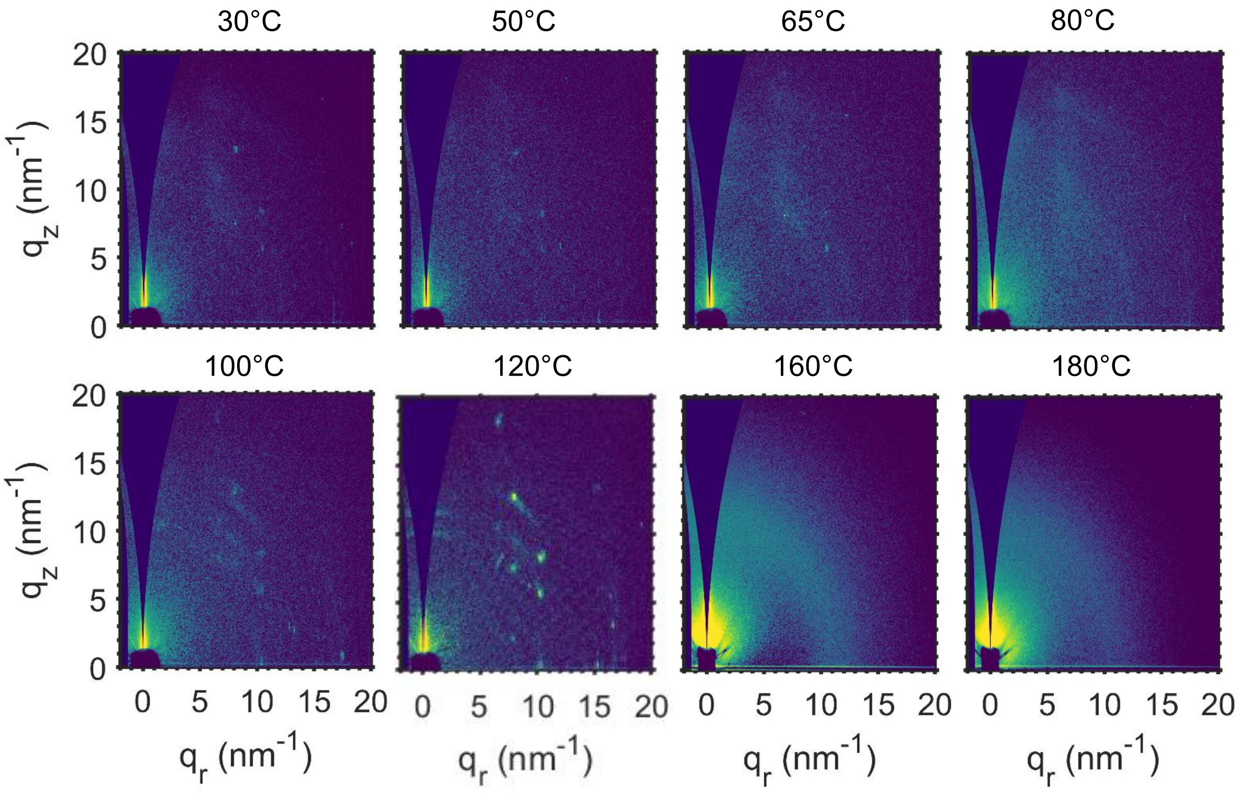


Figure S12: 2D-GIWAXS patterns of films cast from anisole, annealed in glovebox for 5 min at the temperature indicated.

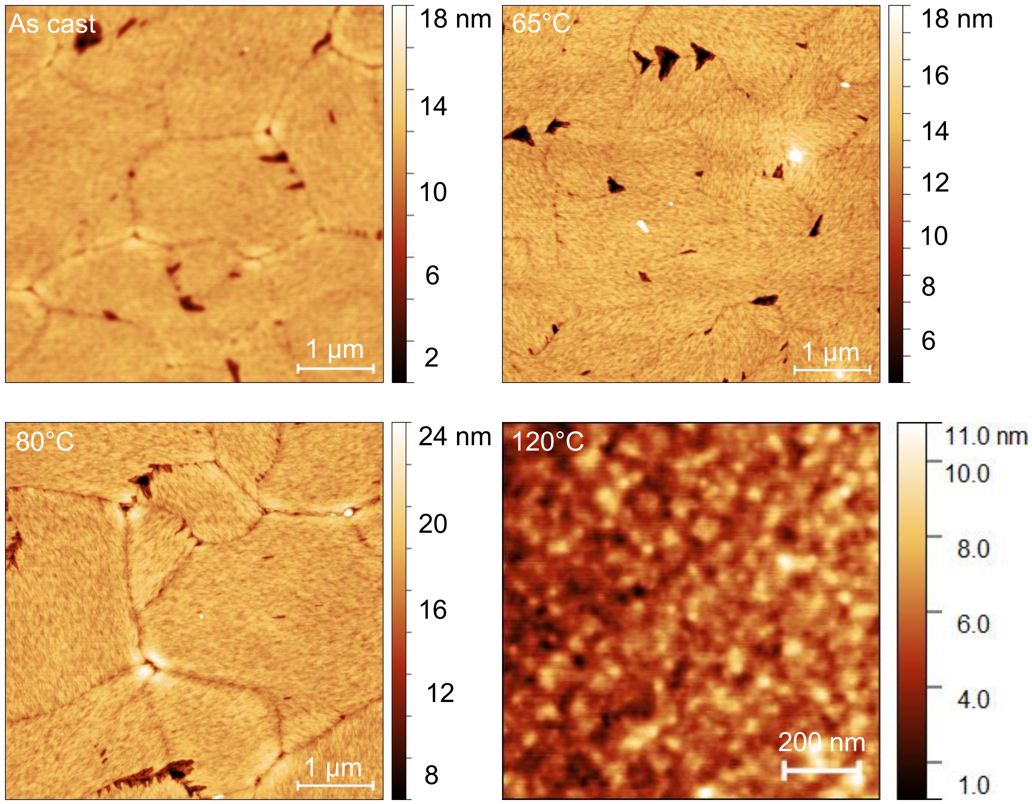


Figure S13: AFM images of β-carotene thin-films cast from anisole annealed at temperatures as indicated.

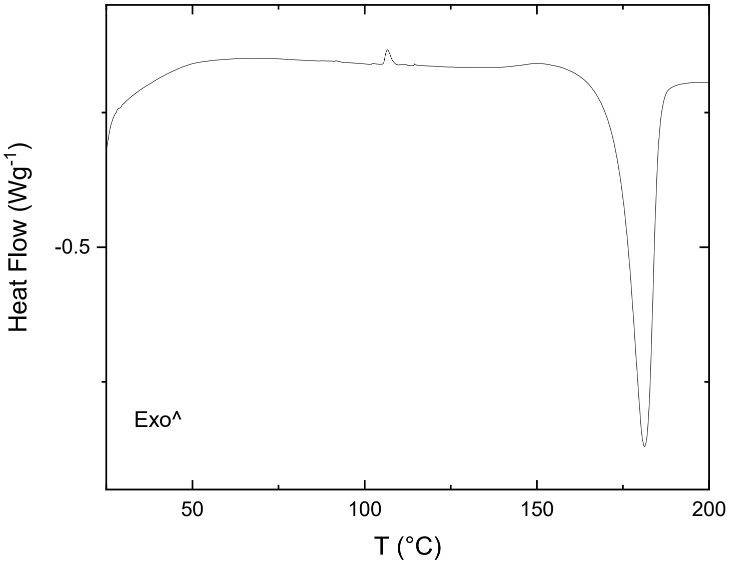


Figure S14: Differential Scanning Calorimetry (DSC) of anisole-processed β-carotene thermographs

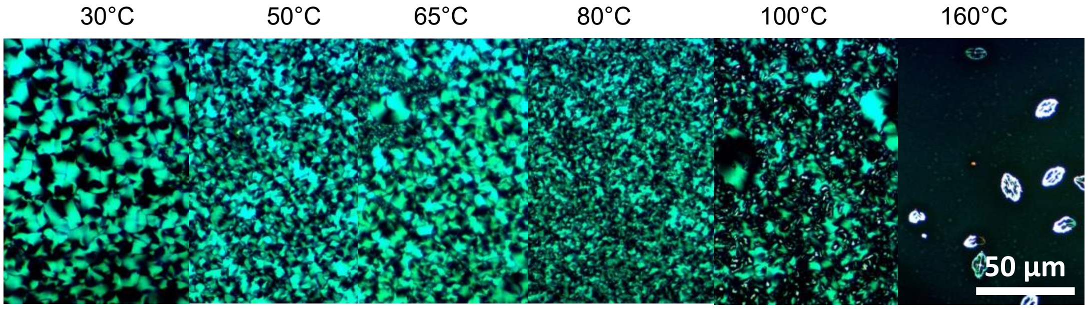


Figure S15: Optical micrographs under polarized light of anisole-processed β-carotene films as a function of annealing temperature.

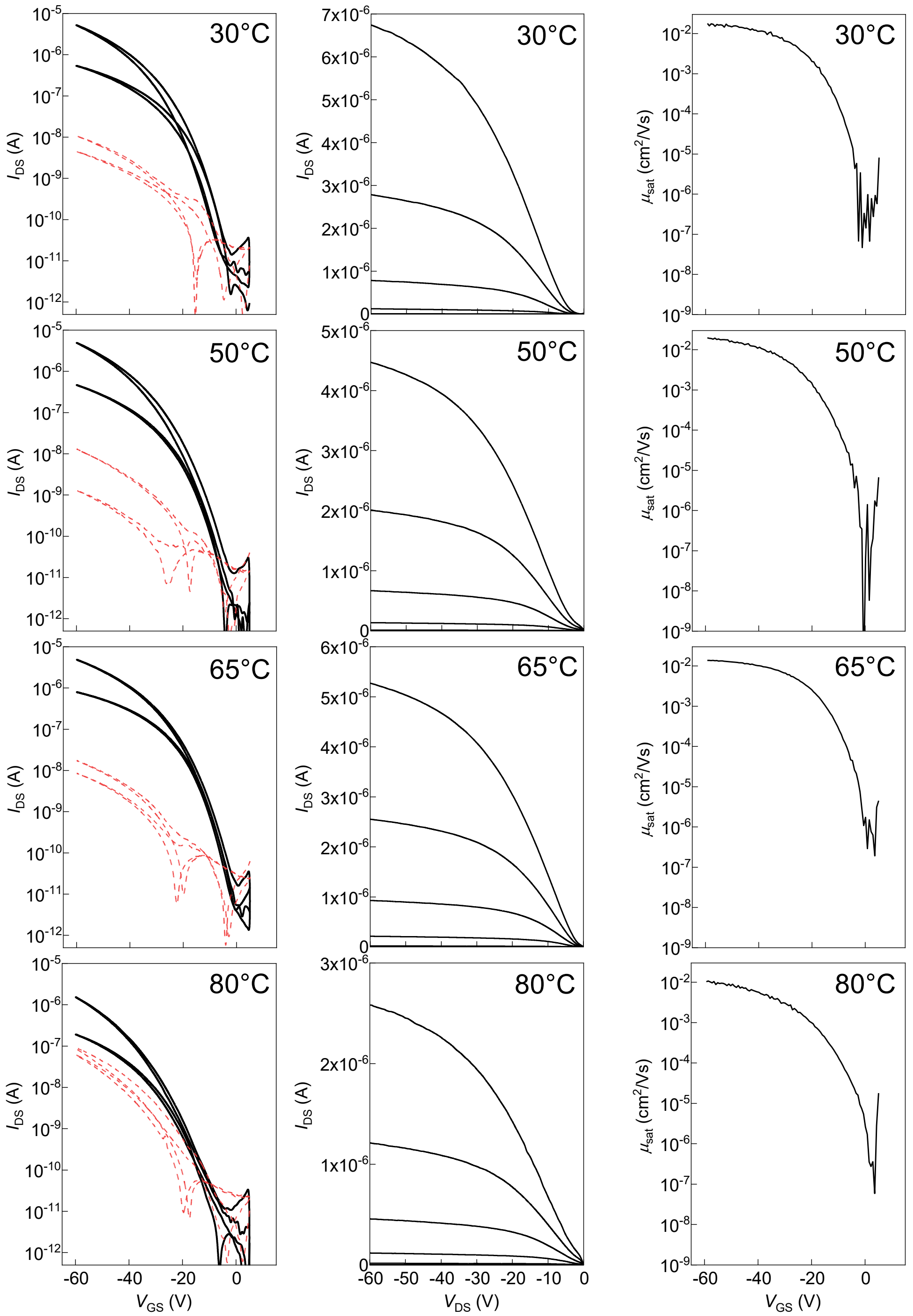


Figure S16: Representative transfer and output characteristics, along with charge-carrier mobility extracted in the saturation regime, for Organic Field-Effect Transistors (OFETs) based on β-carotene films processed from anisole.

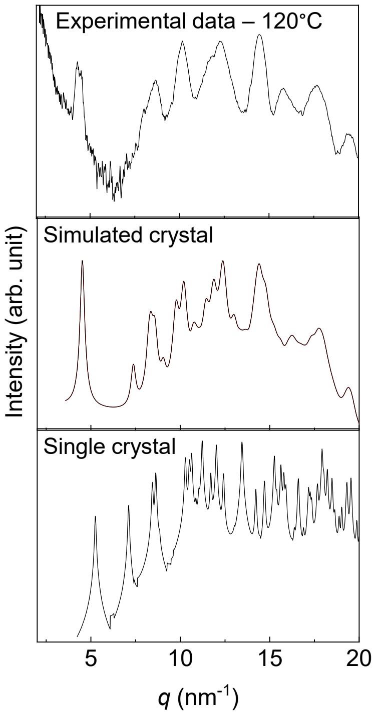


Figure S17: (top) azimuthal integration of GIWAXS pattern of THF processed β-carotene thin-film annealed at 120°C, (middle) refined XRD pattern and (bottom) XRD pattern derived from the single crystal data.

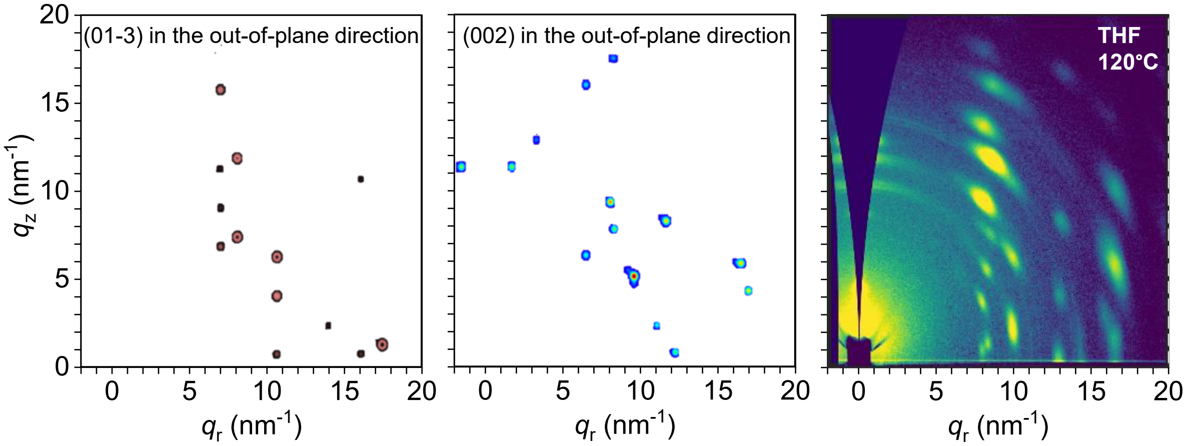


Figure S18: simulated q-maps of the refined structure oriented along the (0 1 –3) and (0 0 2) and the reference 2D-GIWAXS pattern of the THF-processed β-carotene thin-film annealed at 120°C.

## Extended DFT theoretical methods

The properties of β-carotene, pentacene, rubrene and dioctyl[1]benzothieno[3,2-b][1]benzothiophene (C8-BTBT) have been investigated using a two-step protocol, rooted on density functional theory. Crystal unit cells of all systems have been first optimized in periodic boundary conditions in a plane-wave/pseudopotential framework using the Quantum ESPRESSO suite of programs.[1,2] Geometry optimizations have been performed using the unscreened hybrid van der Waals vdW-DF-cx0 functional, particularly suitable for the simulation of large non-covalent organic crystals.[3] Nuclei and core electrons have been substituted by optimized norm-conserving

Vanderbilt pseudopotentials[4] obtained from the SG15 database.[5] Satisfactorily converged results have been obtained using 90/120/360 Ry cutoffs for the representation of wave functions, Fock integrals, and electron density, respectively.

Monomers of all the molecules, as well as dimers chosen along selected crystallographic directions, have been extracted from the optimized crystal structures and investigated in an all-electron/localized basis set framework using the ORCA suite of programs.[6,7] Monomers have been fully optimized in their neutral and cationic configurations to calculate internal reorganization energies for holes, $\lambda^+$, using equation (2)

$$\lambda^+ = \left(E^+\left(M_0\right) - E^+\left(M^+\right)\right) + \left(E^0\left(M^+\right) - E^0\left(M_0\right)\right) \qquad (2)$$

as detailed elsewhere,[8,9] where $E^0(M_0)$ and $E^+(M^+)$ are the total energies of the neutral species and cation in their equilibrium structures, respectively, while $E^+(M_0)$ is the total energy of the cation in the neutral geometry and $E^0(M^+)$ is the total energy of the neutral in the cation geometry. Transfer integrals J have been calculated using the Energy splitting in dimers (ESD) methods, corrected by intermolecular overlap in the Koopmans approximation using equation (3)

$$J \approx \frac{\Delta E}{2}\frac{1-S^2}{1+S^2} \qquad (3)$$

as detailed elsewhere,[10] using nearest-neighbor dimers along all non-equivalent crystallographic directions, extracted from optimized crystal structures and not optimized further. In equation (3), ΔE is the energy difference between the HOMO and HOMO-1 orbitals, and S is the intermolecular overlap integral between the HOMO orbitals of the two monomers, calculated from B3LYP wavefunctions using the Multiwfn program.[11]

Two series of DFT simulations have been performed using the B3LYP[12] and M06-2X[13] functionals in conjunction with all-electron def2-TZVPP basis sets.[14,15] The corresponding def2/J basis has also been used as an auxiliary basis set for Coulomb fitting in a resolution-of-identity/chain-of-spheres (RIJCOSX) framework for the calculation of Coulomb and exchange integrals implemented in ORCA. All the systems have been embedded in an implicit dielectric environment using a conductor-like polarizable continuum model (CPCM).[16] To compare DFT results with accurate wavefunction-based calculations, transfer integrals J have been also calculated using an equation-of-motion coupled-cluster method[17] including single and double excitations (EOMIP-CCSD), using the obtained ionization energies as accurate proxies for HOMO and HOMO-1 in equation (2).[18] Within the ORCA implementation of CCSD, ionization energies are calculated by grouping electron pairs in domain-local pairs of natural orbitals (DLPNO), and the chain-of-sphere approximation is used to speed up the calculation of exchange-like integrals with four virtual labels (COSX).[19]

Finally, time-dependent DFT simulations summarized in Figure S10 have been performed to assess the optical properties of β-carotene films and to compare theoretical results with the evolution of absorption spectra discussed in the main text. Aggregation has been simulated not only using the same dimers selected to calculate transfer integral along the 010, 100 and 101 directions of the in the β-carotene crystal. Amorphous β-carotene structures have been approached by simulating the most stable dimers found by means of a conformer-rotamer ensemble sampling tool (CREST)[20] which uses the xTB-GFN2 Hamiltonian as engine to calculate total energies and forces.[21] TDDFT absorption spectra of β-carotene monomers and dimers have been calculated using the same basis sets discussed above and the range-separated hybrid ωB97X functional.[22] A large basis of 600 (1200) vectors

connecting occupied and unoccupied eigenstates has been used to build the Davidson expansion space for the calculations of the first 30 (60) electronic transitions of monomer (dimers).

**Table S2.** HOMO and LUMO energies and internal reorganization energies for holes, $\lambda^+$, calculated at DFT level using the B3LYP (red) and M062X (blue) functionals.

| **B3LYP**<br>**M062X** | **HOMO (eV)** | **LUMO (eV)** | **$\lambda^+$ (eV)** |
|---|---|---|---|
| **β-carotene** | -4.58<br>-5.90 | -2.38<br>-1.67 | 0.31<br>0.51 |
| **pentacene** | -4.83<br>-5.99 | -2.64<br>-2.05 | 0.10<br>0.17 |
| **rubrene** | -5.02<br>-6.24 | -2.42<br>-1.84 | 0.15<br>0.21 |
| **c8-btbt-c8** | -5.56<br>-6.87 | -1.26<br>-0.54 | 0.24<br>0.30 |

**Table S3.** HOMO and HOMO-1 energies of molecular dimers interacting along the indicated crystallographic directions and corresponding transfer integrals J, calculated using equation (3). The calculations have been carried out at DFT level using the B3LYP (red) and M062X (blue) functionals and at the EOMIP-CCSD level (green). J values larger than 0.01 eV are printed in boldface.

| **B3LYP**<br>**M062X**<br>**EOMIP-CCSD** | **HOMO** | **HOMO-1** | **J (eV)** |
|---|---|---|---|
| **β-carotene** | | | |
| 010 | -4.359<br>-5.472<br>-5.905 | -4.460<br>-5.601<br>-6.050 | **0.050**<br>**0.064**<br>**0.072** |
| 100 | -4.442<br>-5.558 | -4.445<br>-5.561 | 0.002<br>0.002 |

| | | | |
|---|---|---|---|
| 101 | -4.449<br>-5.566 | -4.452<br>-5.569 | 0.002<br>0.002 |
| **pentacene** | | | |
| 010 | -4.684<br>-5.800<br>-6.223 | -4.900<br>-6.047<br>-6.504 | **0.108**<br>**0.123**<br>**0.140** |
| 100 | -4.765<br>-5.890 | -4.837<br>-5.973 | **0.036**<br>**0.042** |
| 110 | -4.722<br>-5.844 | -4.865<br>-6.005 | **0.071**<br>**0.080** |
| -1-11a | -4.811<br>-5.926 | -4.813<br>-5.928 | 0.001<br>0.001 |
| -1-11b | -4.813<br>-5.928 | -4.816<br>-5.931 | 0.002<br>0.002 |
| **rubrene** | | | |
| 001 | -4.973<br>-6.161 | -5.007<br>-6.201 | **0.017**<br>**0.020** |
| 010 | -4.859<br>-6.035<br>-6.215 | -5.055<br>-6.277<br>-6.498 | **0.098**<br>**0.121**<br>**0.142** |
| 100 | -5.004<br>-6.186 | -5.007<br>-6.190 | 0.002<br>0.002 |
| **c8-btbt-c8** | | | |
| 010 | -5.441<br>-6.748<br>-7.149 | -5.555<br>-6.876<br>-7.283 | **0.057**<br>**0.064**<br>**0.067** |

| | | | |
|---|---|---|---|
| 100 | -5.423<br>-6.732 | -5.429<br>-6.745 | 0.003<br>0.007 |
| 110 | -5.529<br>-6.837 | -5.566<br>-6.880 | **0.019**<br>**0.022** |

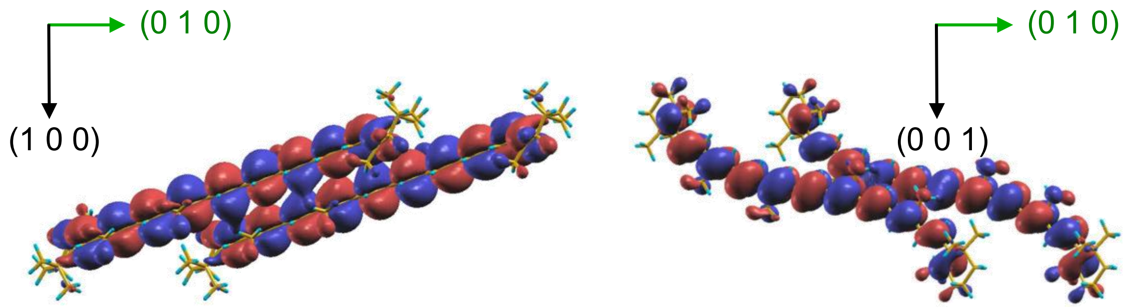


Figure S19: DFT-computed highest occupied molecular orbital (HOMO) for the dimer from different molecular orientations.

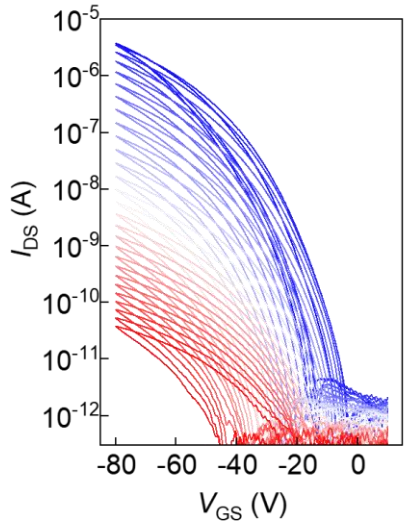


Figure S20: Stability of β-carotene cast from anisole, annealing temperature 65 °C. Transfer characteristics of anisole casted OFETs measured in air as a function of time. Devices were measured every 30 min for 18 h.

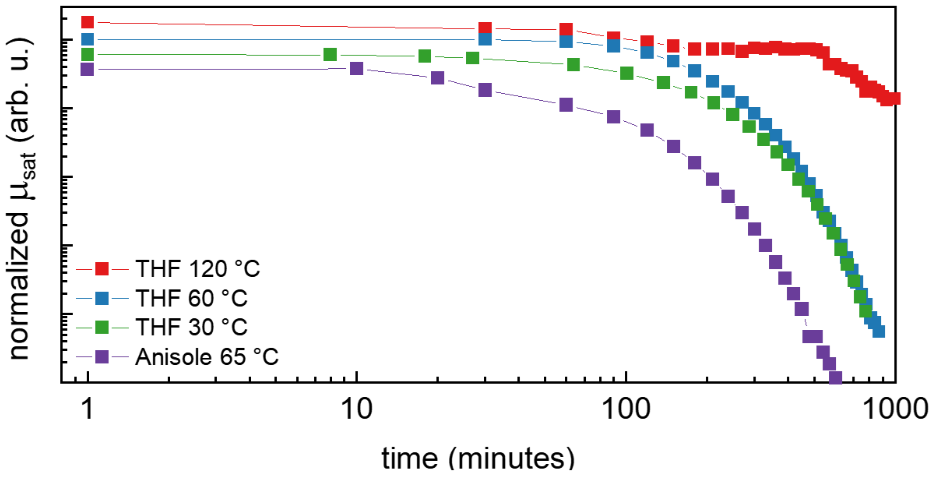


Figure S21: Time evolution of the charge carrier mobility of the OFET from THF (annealing temperatures of 30 °C, 60 ° C, and 120 °C) and anisole (annealing temperature 65 °C). Devices were measured every 30 min for 18 h. The values are normalized to the initial value obtained before exposure to air.

**References**


1 P. Giannozzi, O. Andreussi, T. Brumme, O. Bunau, M. Buongiorno Nardelli, M. Calandra, R. Car, C. Cavazzoni, D. Ceresoli, M. Cococcioni, N. Colonna, I. Carnimeo, A. Dal Corso, S. de Gironcoli, P. Delugas, R. A. DiStasio, A. Ferretti, A. Floris, G. Fratesi, G. Fugallo, R. Gebauer, U. Gerstmann, F. Giustino, T. Gorni, J. Jia, M. Kawamura, H.-Y. Ko, A. Kokalj, E. Küçükbenli, M. Lazzeri, M. Marsili, N. Marzari, F. Mauri, N. L. Nguyen, H.-V. Nguyen, A. Otero-de-la-Roza, L. Paulatto, S. Poncé, D. Rocca, R. Sabatini, B. Santra, M. Schlipf, A. P. Seitsonen, A. Smogunov, I. Timrov, T. Thonhauser, P. Umari, N. Vast, X. Wu and S. Baroni, *Journal of Physics: Condensed Matter*, 2017, 29, 465901.

2 P. Giannozzi, S. Baroni, N. Bonini, M. Calandra, R. Car, C. Cavazzoni, D. Ceresoli, G. L. Chiarotti, M. Cococcioni, I. Dabo, A. Dal Corso, S. de Gironcoli, S. Fabris, G. Fratesi, R. Gebauer, U. Gerstmann, C. Gougoussis, A. Kokalj, M. Lazzeri, L. Martin-Samos, N. Marzari, F. Mauri, R. Mazzarello, S. Paolini, A. Pasquarello, L. Paulatto, C. Sbraccia, S. Scandolo, G. Sclauzero, A. P. Seitsonen, A. Smogunov, P. Umari and R. M. Wentzcovitch, *Journal of Physics: Condensed Matter*, 2009, 21, 395502.

3 K. Berland, Y. Jiao, J.-H. Lee, T. Rangel, J. B. Neaton and P. Hyldgaard, *J Chem Phys*, DOI:10.1063/1.4986522.

4 D. R. Hamann, *Phys Rev B*, 2013, 88, 085117.

5 M. Schlipf and F. Gygi, *Comput Phys Commun*, 2015, 196, 36–44.

6 F. Neese, *WIREs Computational Molecular Science*, DOI:10.1002/wcms.1327.

7 F. Neese, *WIREs Computational Molecular Science*, 2012, 2, 73–78.

8 R. Oshi, S. Abdalla and M. Springborg, *The European Physical Journal D*, 2019, 73, 124.

9 M. S. Stark, *J Phys Chem A*, 1997, 101, 8296–8301.

10 V. Coropceanu, J. Cornil, D. A. da Silva Filho, Y. Olivier, R. Silbey and J.-L. Brédas, *Chem Rev*, 2007, 107, 926–952.

11 T. Lu and F. Chen, *J Comput Chem*, 2012, 33, 580–592.

12 A. D. Becke, *J Chem Phys*, 1993, 98, 5648–5652.

13 Y. Zhao and D. G. Truhlar, *Theor Chem Acc*, 2008, 120, 215–241.

14 A. Schäfer, H. Horn and R. Ahlrichs, *J Chem Phys*, 1992, 97, 2571–2577.

15 F. Weigend and R. Ahlrichs, *Physical Chemistry Chemical Physics*, 2005, 7, 3297.

16 V. Barone and M. Cossi, *J Phys Chem A*, 1998, 102, 1995–2001.

17 R. J. Bartlett, *WIREs Computational Molecular Science*, 2012, 2, 126–138.

18 A. Pershin and P. G. Szalay, *J Chem Theory Comput*, 2015, 11, 5705–5711.

19 A. K. Dutta, F. Neese and R. Izsák, *J Chem Phys*, DOI:10.1063/1.4939844.

20 P. Pracht, F. Bohle and S. Grimme, *Physical Chemistry Chemical Physics*, 2020, 22, 7169–7192.

21 C. Bannwarth, S. Ehlert and S. Grimme, *J Chem Theory Comput*, 2019, 15, 1652–1671.

22 J.-D. Chai and M. Head-Gordon, *J Chem Phys*, DOI:10.1063/1.2834918.
